# High-throughput production of cheap mineral-based two-dimensional electrocatalysts for high-current-density hydrogen evolution

**DOI:** 10.1038/s41467-020-17121-8

**Published:** 2020-07-24

**Authors:** Chi Zhang, Yuting Luo, Junyang Tan, Qiangmin Yu, Fengning Yang, Zhiyuan Zhang, Liusi Yang, Hui-Ming Cheng, Bilu Liu

**Affiliations:** 1 Shenzhen Geim Graphene Center, Tsinghua-Berkeley Shenzhen Institute & Tsinghua Shenzhen International Graduate School, Tsinghua University, Shenzhen, 518055 P. R. China; 2 Shenyang National Laboratory for Materials Sciences, Institute of Metal Research, Chinese Academy of Sciences, Shenyang, Liaoning 110016 P. R. China

**Keywords:** Catalyst synthesis, Electrocatalysis, Two-dimensional materials

## Abstract

The high-throughput scalable production of cheap, efficient and durable electrocatalysts that work well at high current densities demanded by industry is a great challenge for the large-scale implementation of electrochemical technologies. Here we report the production of a two-dimensional molybdenum disulfide-based ink-type electrocatalyst by a scalable exfoliation technique followed by a thermal treatment. The catalyst delivers a high current density of 1000 mA cm^−2^ at an overpotential of 412 mV for the hydrogen evolution. Using the same method, we produce a cheap mineral-based catalyst possessing excellent performance for high-current-density hydrogen evolution. Noteworthy, production rate of this catalyst is one to two orders of magnitude higher than those previously reported, and price of the mineral is five orders of magnitude lower than commercial Pt electrocatalysts. These advantages indicate the huge potentials of this method and of mineral-based cheap and abundant natural resources as catalysts in the electrochemical industry.

## Abstract (web summary)

The large-scale implementation of electrochemical technologies will require the high-throughput production of high-performance, inexpensive catalysts. Here, authors demonstrate earth abundant molybdenite as raw materials to produce efficient MoS_2_ catalysts for high current density H_2_ evolution.

## Introduction

The large-scale production of hydrogen by electrochemical water splitting has been proposed as a promising technology for a sustainable energy source because water is abundant, sustainable, and carbon-free, and the electricity for the hydrogen production can be generated by wind and sunlight^1,2^. However, the energy consumption in the hydrogen evolution reaction (HER, 2H^+^ + 2e^−^ → H_2_) is usually high due to its slow reaction kinetics, resulting in the need for efficient and durable electrocatalysts^3^. Platinum (Pt) is the most efficient electrocatalyst for HER, but its abundance is six orders of magnitude lower than aluminum, the most abundant metal^4,5^, making it extremely expensive (ten million US$ per ton, Supplementary Fig. 1). As a result, the Pt or any other noble metal catalyst accounts for ~8% of the total stack cost of a proton exchange membrane electrolyzer^6^. Because of this reason, researchers have devoted great effort to exploring Pt-free or low-Pt electrocatalysts, including Pt single atoms^7,8^, Pt-based alloys^9,10^, metal carbides^11–13^, transition metal dichalcogenides (TMDCs)^14–20^, metal phosphides^6,21^, etc. For example, Liu et al. anchored Pt single atoms on carbon nanospheres, and the resulting material showed a comparable HER performance to commercial Pt/C but with less Pt needed^7^. King et al. loaded CoP nanoparticles on a high-surface-area carbon support, which showed an excellent activity and a long-term stability that were close to commercial Pt/C^6^. Although great progress has been made in the low-Pt and Pt-free electrocatalysts, how to achieve the high-throughput production of such catalysts is still a challenge to use water electrolysis. Moreover, the current densities needed by industry are usually higher than 1000 and 500 mA cm^−2^ for proton exchange membrane and alkaline electrolyzers^13^, requiring catalysts with good electrochemical, thermal, and mechanical stabilities, as well as abundant numbers of active sites^6,13,22^. These challenges have motivated the need for the high throughput production of efficient, durable, and cheap electrocatalysts for a high-current-density HER.

Molybdenum disulfide (MoS_2_) is promising for HER because of its high catalytic activity, good stability, and low price. Hinnemann et al. forecast that MoS_2_ edges would be active for HER with a free adsorption energy of hydrogen close to zero^23^, which was then experimentally verified by Jaramillo et al. using two-dimensional (2D) MoS_2_^15^ because the 2D form had more exposed edges than the bulk material^16,24,25^. Our group has recently demonstrated that by combining surface chemistry and morphology engineering, MoS_2_ showed a good HER performance at current density of 1000 mA cm^−2^ ^13^. MoS_2_ also has a good atmospheric thermal stability (up to 300 °C)^26^, a good electrochemical stability under reducing potentials^26^, and is mechanically roubust^27,28^, making it a promising catalyst for high-current density HER. The global proven reserve of Mo is about 500 times that of Pt and its price is three orders of magnitude lower (Supplementary Fig. 1), making the cheap production of MoS_2_-based catalysts possible. MoS_2_ naturally exists as the molybdenite mineral, with a global availability of 17,000,000 ton^4^. If one can use such a low-cost and abundant mineral to produce suitable catalysts, the overall cost of HER electrocatalysts would be significantly reduced. Despite its good performance and stability, most of the methods to produce MoS_2_-based catalysts are energy intensive and/or difficult to scale-up due to the use of conditions such as high vacuum^15^, poisonous reactants^24,25^, high pressure^13,26^, or the poor high-current-density HER performance. Therefore, the production of efficient, cheap, and durable MoS_2_-based catalysts by a high-throughput and scalable way is desired to make a real impact in HER electrochemical technology.

Here, we report a high-throughput scalable method for production of cheap yet high-performance MoS_2_-based HER catalysts that work well at high current densities up to 1000 mA cm^−2^. We first obtain 2D MoS_2_ flakes by a scalable top-down exfoliation method, followed by a simple thermal treatment to prepare the catalyst, with both processes having the possibility of being scaled up for high-throughput production. The catalysts are 2D MoS_2_ modified by Mo_2_C nanoparticles on their edges and surfaces, and have a good HER performance with a high current density of 1000 mA cm^−2^ at 412 mV, a small Tafel slope of 60 mV dec^−1^, and good stability for 24 h. We also demonstrate the feasibility of the high-throughput production method by using a cheap molybdenite concentrate from a naturally existing earth-abundant mineral and find that the mineral catalysts also show good HER performance at high current densities. The production rate of the electrocatalyst is as high as 1.3 g h^−1^, one to two orders of magnitude higher than previous results (Supplementary Table 1), and the catalyst price is ~10 US$ m^−2^, around 30 times lower than than a commercial Pt/C electrocatalyst.

## Results

### Preparation of MoS_2_-based ink-type electrocatalysts

**Fig. 1:**
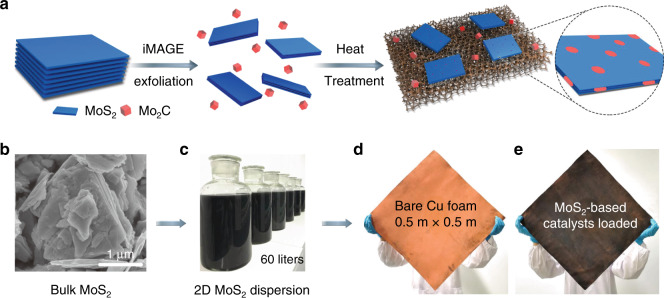
High-throughput production of molybdenum disulfide (MoS_2_)-based ink-type electrocatalysts. **a** Schematic of the fabrication method of MoS_2_-based catalysts. **b** Scanning electron microscopy image of the pristine MoS_2_ powder, and photos of (**c**) two-dimensional (2D) MoS_2_ aqueous dispersion with a volume of 60 liters, (**d**) bare copper (Cu) foam and (**e**) MoS_2_-based catalyst loaded on Cu foam.

A schematic of the process is shown in Fig. 1a. The MoS_2_-based catalyst was synthesized by a two-step method, i.e., exfoliation of bulk MoS_2_ into 2D flakes followed by thermal treatment (see details in the “Methods” section). In brief, 2D MoS_2_ was first prepared by exfoliating bulk MoS_2_ (Supplementary Fig. 2) by an interMediate-Assisted Grinding Exfoliation (iMAGE) technique that was able to obtain 2D materials at the tonne scale^28^. Here we used a modified iMAGE technique that used Mo_2_C as the force intermediary to facilitate the exfoliation of MoS_2_ because it has a low electrical resistivity (57  μΩ cm) and a high hardness (Moh’s hardness is 7)^29^. The 2D MoS_2_ flakes were then dispersed in water to obtain a catalyst dispersion, which is ink-type and is suitable to be integrated with robust industrial used techniques to produce large-area electrodes such as dip-coating, drop-casting, roll-to-roll printing, screen printing, and spray coating. Here, the exfoliated MoS_2_ flakes together with Mo_2_C were loaded by dip coating onto supports with high surface areas (e.g., carbon cloth, Ti substrate, Cu foam) for the CH_4_/H_2_ thermal treatment, after which 2D MoS_2_ became a suitable catalyst for the subsequent HER test (denoted as HC-MoS_2_/Mo_2_C, where “HC” means CH_4_/H_2_ thermal treatment). To optimize the HER performance of the catalyst, the thermal treatment was divided into two parts, i.e., first the desulfurization using H_2_ as the reacting gas to form S vacancies on 2D MoS_2_, and second the carburization using CH_4_/H_2_ as the reacting gases to produce Mo_2_C nanoparticles on 2D MoS_2_^13^. In the first stage, S vacancies were formed by the reaction of H_2_ with S in the MoS_2,_ removing S atoms to form H_2_S, while in the second stage CH_4_ reacted with Mo atoms near the vacancies to form Mo_2_C by dehydrogenation^30^. Both processes involved mild conditions and could be scaled-up for the high-throughput production of MoS_2_-based catalysts. Examples of several MoS_2_-based catalysts on different supports prepared by this method are shown in Fig. 1b–d and S3.

### Structure characterization of the catalysts

**Fig. 2:**
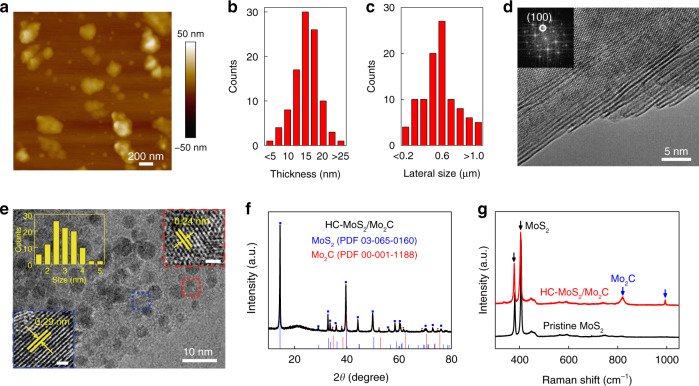
Material characterization. **a** Atomic force microscopy image and statistical analysis of (**b**) the lateral size and **c** the thickness of the 2D MoS_2_ flakes. **d** High resolution transmission electron microscopy (HRTEM) image of the 2D MoS_2_. Inset is the corresponding fast Fourier transform (FFT) pattern. **e** HRTEM image of the HC-MoS_2_/Mo_2_C. The insets are a histogram of the lateral size of the Mo_2_C nanocrystals, and two high magnification HRTEM images of a MoS_2_ flake (the blue dotted box) and a Mo_2_C nanocrystal (the red dotted box). The scale bars in the insets are 1 nm. **f** X-ray diffraction pattern and (**g**) Raman spectra of the 2D MoS_2_ and the HC-MoS_2_/Mo_2_C.

We characterized the structures of the MoS_2_ materials after the first exfoliation step and the second thermal treatment step. To expose more MoS_2_ edges, we choose a bulk MoS_2_ material with a relatively small lateral size (~1 μm). Atomic force microscopy (AFM) showed that after the first exfoliation step, the bulk MoS_2_ was exfoliated into 2D MoS_2_ flakes (Fig. 2a) with an average thickness of 15 nm (Fig. 2b) and an average lateral size of 0.6 μm (Fig. 2c), consistent with the results of dynamic light scattering (DLS, Supplementary Fig. 4), transmission electron microscopy (TEM, Supplementary Fig. 5), and scanning electron microscopy (SEM, Supplementary Fig. 5). The uniformity of the 2D MoS_2_ was demonstrated by combined AFM, SEM, and TEM characterization. In addition, results from high resolution transmission electron microscopy (HRTEM) and the corresponding fast Fourier transformation (FFT) showed the high quality of the 2D MoS_2_ flakes without noticeable defects in their basal planes and edges (Fig. 2d and Supplementary Fig. 6). The above results show that in the first step the bulk MoS_2_ was exfoliated into 2D MoS_2_ flakes with good uniformity and high quality.

After the second thermal treatment step, we confirmed the formation of Mo_2_C nanocrystals on the 2D MoS_2_ by HRTEM, X-ray diffraction (XRD), and Raman spectroscopy. The HRTEM images show that in the final optimized HC-MoS_2_/Mo_2_C catalyst, *α*-Mo_2_C nanocrystals were formed both on the basal planes and edges of 2D MoS_2_ flakes (Fig. 2e and Supplementary Fig. 7). The growth of Mo_2_C nanocrystals in these positions is easy to understand because S vacancies and unsaturated Mo existed there, which facilitated their formation. Higher magnification HRTEM images show typical lattice spacings of 0.29 nm and 0.24 nm, which, respectively, correspond to the (100) planes of MoS_2_ (blue dotted box in Fig. 2e) and the (002) planes of *α*-Mo_2_C (red dotted box in Fig. 2e). Statistical analysis shows that the Mo_2_C nanocrystals (inset of Fig. 2e) have an average diameter of 2.8 nm and a narrow size distribution, and are evenly distributed over the 2D MoS_2_ basal planes. Regarding the XRD patterns (Fig. 2f and Supplementary Fig. 8), the MoS_2_ (002) peak is quite sharp, indicating the high crystallinity of the MoS_2_. The peaks at 34.5° and 38.1° correspond to the (100) and (002) planes of Mo_2_C. Moreover, Raman spectroscopy was also used to identify the structures of the HC-MoS_2_/Mo_2_C (Fig. 2g). In addition to the MoS_2_ peaks, those at 812 and 987 cm^−1^ indicate the formation of Mo_2_C^31^. Note that a lateral heterostructure made of MoS_2_/*α*-Mo_2_C has been constructed. Taking these results together, we can say that the HC-MoS_2_/Mo_2_C is composed of 2D MoS_2_ flakes with a large number of Mo_2_C nanocrystals on their basal planes and edges, and good uniformity of the catalyst is achieved by this method.

### HER performance at high current densities

**Fig. 3:**
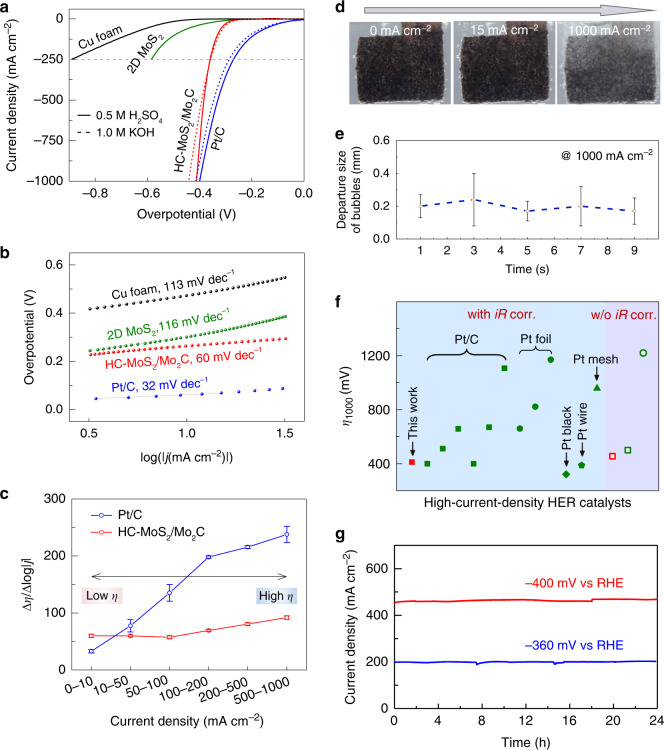
Electrocatalytic performance of different catalysts for high-current-density HER. **a** Polarization curves, (**b**) Tafel plots, and (**c**) Δ*η*/Δlog|*j* | ratios of different catalysts in 0.5 M H_2_SO_4_ at a scan rate of 5 mV s^−1^ with *iR* correction. **d** Photos and (**e**) corresponding size distribution of H_2_ bubbles leaving the surfaces of HC-MoS_2_/Mo_2_C catalysts. **f** A comparison of the HER performance of HC-MoS_2_/Mo_2_C catalysts and Pt-based catalysts we tested and previously reported (see details in Supplementary Table 2). The “w/o *iR* corr.” and “with *iR* corr.” in (**f**) mean without and with *iR* correction. **g** Chronoamperometric response (*i*–*t*) curves for hydrogen evolution reaction (HER) using HC-MoS_2_/Mo_2_C at current densities of 200 and ~500 mA cm^−2^ for 24 h, corresponding to potentials of −360 and −400 mV *vs* reversible hydrogen electrode (RHE) without *iR* correction. The error bars show the standard derivation in (**c**) and (**d**).

To evaluate the electrochemical performance of the catalysts, we used a standard three-electrode electrolyzer. All the electrochemical characterization was conducted using the same cell and the same test parameters (Supplementary Fig. 9). The distance between the working and reference electrodes was optimized to measure the HER performance at high current densities and the catalysts were tailored to ensure identical electrode surface areas (Supplementary Fig. 10). The HER performance of catalysts was compared by the overpotential (*η*) as given in the following equation:

1 $$\eta = {\it{E}}_{{\it{{\rm{appl}}}}} - {\it{E}}_{{\it{{\rm{eq}}}}} - {\it{{\rm{i}}R}}_{{\rm{s}}},$$

where ${\it{E}}_{{\rm{appl}}}$ and ${\it{E}}_{{\rm{eq}}}$ are the practical applied and theoretical electrochemical equilibrium potentials (0 V vs RHE for HER), and ${{{\rm{i}}R}}_{{{\rm{s}}}}$ is the solution resistance drop. Several key parameters such as the nature of the supports, the loading amount of 2D MoS_2_ flakes, as well as the temperature and time for the desulfurization and carburization processes have been systematically studied and optimized (Supplementary Figs. 3, 11, and 12). Based on the best samples obtained, we characterized and compared the HER performance of the HC-MoS_2_/Mo_2_C with other samples, a series of Pt-based catalysts, bare Cu foam, and 2D MoS_2_ without the thermal treatment. Polarization curves of these samples in a H_2_SO_4_ (0.5 M) solutions are shown in Fig. 3a, Supplementary Fig. 13 and Movie 1. The dissolution of Cu in sulfuric acid and the effect of Cu support on current densities in HER is negligible in our experiments (Supplementary Fig. 14). Compared to the best Pt sample with morphology engineering, i.e., Pt/C loaded on high-surface-area Cu foam with a loading mass of 2 mg cm^−2^, the HC-MoS_2_/Mo_2_C needed a larger overpotential to obtain an identical current density (*j*) at small current densities, suggesting that it has a lower intrinsic activity than Pt (Supplementary Fig. 15 and 16). Tafel plots were used to study the rate-determining step of the catalysts (Fig. 3b) and the results show that the HC-MoS_2_/Mo_2_C has a small slope of 60 mV dec^−1^, indicating that the recombination of hydrogen is its rate-limiting step^32^. As current density increased to over 400 mA cm^−2^, the HC-Mo_2_S/Mo_2_C required comparable overpotentials to obtain the same current density to the Pt-based catalysts (Supplementary Fig. 17 and Table 2). For instance, the HC-MoS_2_/Mo_2_C needed 412 mV @ 1000 mA cm^*−*2^, while the respective values for 0.5 mg cm^−2^ Pt/C and 2 mg cm^−2^ Pt/C were 511 mV and 400 mV. These results show the superior HER performance of the HC-MoS_2_/Mo_2_C at high current densities. Moreover, the HC-MoS_2_/Mo_2_C gives a nearly 100% Faradaic efficiency during HER (Supplementary Fig. 18). Neither the 2D MoS_2_ without the thermal treatment nor the bare Cu foam showed such a performance as the current density increased. Note that for the sample without the thermal treatment, the 2D MoS_2_ flakes fell from the support as current density increased and made the catalytic performance even poorer, caused by the forces of H_2_ bubbles (Supplementary Fig. 19 and Movie 2). In sharp contrast, the HC-MoS_2_/Mo_2_C was tightly attached to the Cu foam support and showed good mechanical stability at high current densities (Supplementary Movie 1). An SEM image of the HC-MoS_2_/Mo_2_C showed a soldering-like phenomenon between the HC-MoS_2_/Mo_2_C and the Cu support, indicating that thermal treatment may be a good way to improve the mechanical robustness of electrocatalysts. The soldering effect could not only provide good electrical contact to improve catalytic performance but also enhance the robustness of the electrode, which is not a necessary result achieved during usual annealing or heat treatment. In addition, the HC-MoS_2_/Mo_2_C catalyst showed a pH-universal HER activity and worked well in a 1.0 M KOH solution (Fig. 3a, Supplementary Figs. 20 and 21).

For practical applications, the high-current-density performance of catalysts is of vital importance. To evaluate the HER performance of the HC-MoS_2_/Mo_2_C in such conditions, we analyzed the relationships between current densities and Δ*η*/Δlog|*j*|, which can be viewed as a generalized slope and can be used to evaluate the performance of catalysts at high current densities^13^. The Pt/C showed an increasing Δ*η*/Δlog|*j*| ratio as current density increased, whereas the HC-MoS_2_/Mo_2_C maintained a small ratio at different current densities, indicating that it had an excellent HER performance at high current densities (Fig. 3c). To understand the different performances of the HC-MoS_2_/Mo_2_C and Pt catalysts, their mass transfer abilities were studied. The diameters of the H_2_ bubbles remained small on HC-MoS_2_/Mo_2_C (Fig. 3d), while they became larger on Pt catalyst as current density increased^13^. Moreover, in HC-MoS_2_/Mo_2_C we did not observe the fluctuation phenomenon at high current densities that is seen for Pt foil and Pt mesh (Supplementary Fig. 17), which might be related to the fast removal of H_2_ bubbles from the surface of HC-MoS_2_/Mo_2_C. We confirmed this point by studying the time-dependent (30 frames per second) transport of H_2_ bubbles on HC-MoS_2_/Mo_2_C, which showed the departure diameters of bubbles on its surface remained small (Fig. 3e). These results indicate the good mass transfer ability of HC-MoS_2_/Mo_2_C at high current densities. Besides mass transfer, we measured the electrochemically active surface areas (ECSA) of these catalysts, which showed that the value for HC-MoS_2_/Mo_2_C was higher than for the other samples and was comparable to 2 mg cm^−2^ Pt/C (Supplementary Fig. 16). Electrochemical impedance spectroscopy (EIS) showed that HC-MoS_2_/Mo_2_C had a small charge transfer resistance (Supplementary Fig. 22). Compared with Pt-based catalysts we tested and previously reported, the HC-MoS_2_/Mo_2_C showed one of the best achievable HER performance at 1000 mA cm^−2^ (Fig. 3f, Supplementary Table 2 and 3). In addition, stability tests showed that HC-MoS_2_/Mo_2_C retained its HER performance over 24 h at 200 mA cm^−2^ and also at ~500 mA cm^−2^ (Fig. 3g), which was confirmed by SEM images (Supplementary Fig. 19). All these results show that HC-MoS_2_/Mo_2_C has good achievable HER performance, demonstrated by a low overpotential, a small Tafel slope, and a good stability at high current density.

### Production of cheap MoS_2_ mineral catalysts for HER

**Fig. 4:**
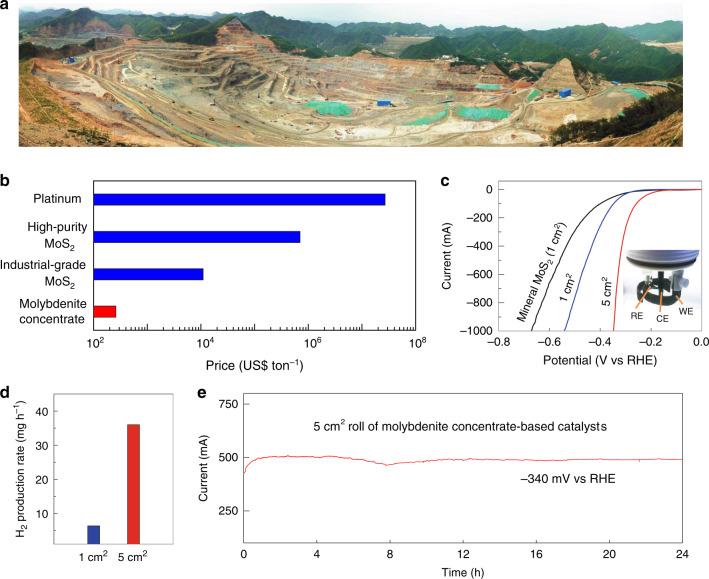
MoS_2_ mineral catalysts from cheap molybdenite concentrates for high-current-density HER. **a** Bird’s eye view of the Sandaozhuang open-pit molybdenite mine in Luoyang, China. Reproduced from Ref. ^28^. The length of the field is around 1.5 km. **b** Commodity price differences between platinum, high-purity MoS_2_, industrial-grade MoS_2_, and molybdenite concentrate. **c** Polarization curves of the different catalysts in 0.5 M H_2_SO_4_ at a scan rate of 5 mV s^−1^ with *iR* correction. Inset is an optical image of a three-electrode electrolyzer for larger working electrodes. **d** H_2_ production rate of different molybdenite concentrate-based catalysts to produce hydrogen under −390 mV *vs* RHE without *iR* correction. **e** Chronoamperometric response (*i*–*t*) curve of a 5 cm^2^ roll of cheap mineral-based catalyst for HER at current of ~500 mA over 24 h.

To realize the large-scale use of electrolyzers, it is important to reduce the cost of all system components, and replacing expensive precious metals with cheap and durable catalysts is a critical step. The high-throughput production of such catalysts can pave the way for their cost reduction as well allowing their use on a large scale. To demonstrate the feasibility of high-throughput production method, we used molybdenite concentrate as the precursor, which is mainly composed of bulk MoS_2_ in form of molybdenite mineral dug directly from an open-pit mine (Fig. 4a). A molybdenite concentrate is generated by a preliminary floatation treatment that can be used to produce industrial-grade MoS_2_ as well as high-purity MoS_2_. Detailed characterization indicated that the molybdenite concentrate mainly composed of crystalline MoS_2_, as well as molybdenum oxides (MoO_2_ and MoO_3_), silicates and few other compositions (Supplementary Figs. 23–25). Note that its current price is only 10^−2^, 10^−4^, 10^−5^ times those of industrial-grade MoS_2_, high-purity MoS_2_, and Pt, respectively (Fig. 4b). It is therefore reasonable to use this raw bulk material to explore industrial catalyst production. A thousand liters of 2D MoS_2_ dispersions (~10 mg mL^−1^) have now been produced in which the 2D MoS_2_ flakes have an average size of 50–100 nm (Supplementary Fig. 26). We have therefore shown that 2D MoS_2_ can be mass produced from a cheap molybdenite concentrate.

We then tested the HER performance of this MoS_2_ mineral-based catalyst. Similar to the earlier experiment, a large amount of 2D MoS_2_ (10 mg mL^−1^) was loaded onto a conductive support followed by thermal treatment. Because this 2D MoS_2_ was mass produced, the size of the working electrode can be made much bigger. We have therefore constructed working electrodes with areas of 1 and 5 cm^2^, both of which showed good uniformity (Supplementary Fig. 27). To the best of our knowledge, the use of such large-scale produced cheap MoS_2_ mineral as HER catalysts have not been reported previously. The difference in the HER performance between the MoS_2_ mineral-based and high-purity MoS_2_-based catalysts has been measured. The MoS_2_ mineral-based catalyst showed a good HER performance at high current densities. For example, it showed a 541 mV @ 1000 mA cm^−2^, only 30% below the value for HC-MoS_2_/Mo_2_C (Supplementary Fig. 28). The good HER performance of the bigger electrode was further demonstrated by EIS (Supplementary Fig. 29), which is quite encouraging because a large amount of HER-inert impurities, e.g., silicates, are present in MoS_2_ concentrates and the catalytic performance can be further improved as discussed in Supplementary Figs. 11 and 12. The polarization curves of different working electrodes in a H_2_SO_4_ (0.5 M) solution are shown in Fig. 4c and S30. They show that the 5 cm^2^ working electrode achieves a current of 1000 mA at 347 mV, which is 194 mV less than for a 1 cm^2^ electrode. We also calculated the H_2_ production rate for different working electrodes (Fig. 4d and Supplementary Table 4) and found that the performance of the 5 cm^2^ electrode (38 mg h^−1^) was an almost perfect scale-up from the 1 cm^2^ electrode (7.4 mg h^−1^), indicating the possibility of industrial use. Furthermore, the MoS_2_ mineral-based catalyst exhibited good durability, maintaining its performance at the current of 500 mA for more than 24 h (Fig. 4e).

**Fig. 5:**
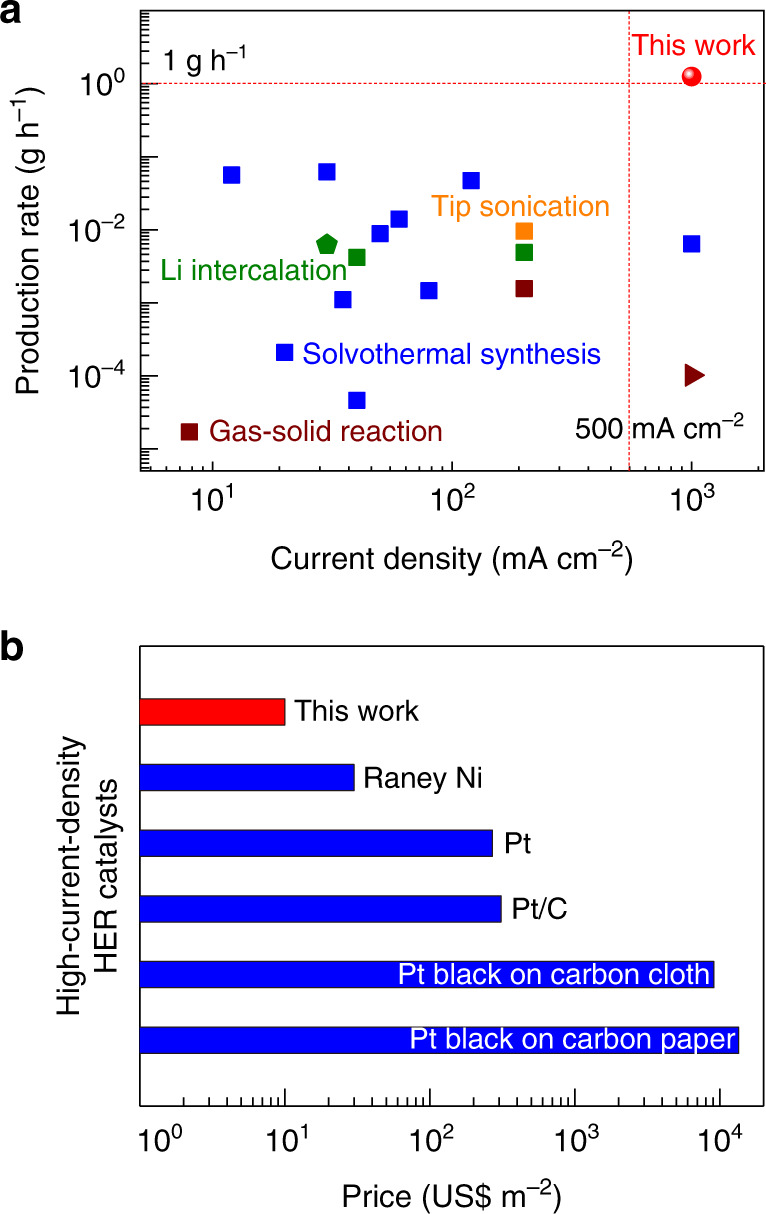
High-efficiency and low-cost production of MoS_2_ mineral-based catalysts from cheap molybdenite concentrate for high-current-density HER. **a** A comparison of the fabrication rate and highest tested current density of transition metal dichalcogenide-based HER catalysts by different methods. Details for data points are shown in Supplementary Table 1. **b** A comparison of the catalyst cost compared to commercial electrocatalysts for HER, showing the ultralow cost of the HC-MoS_2_/Mo_2_C catalyst. Note that the support costs of HC-MoS_2_/Mo_2_C, Raney Ni, Pt and Pt/C catalysts are excluded.

The fabrication method for the HC-MoS_2_/Mo_2_C catalyst has noticeable advantages over other methods such as solvothermal synthesis, gas–solid reaction, tip sonication, and Li intercalation (Fig. 5a and Supplementary Table 1). For example, the production rate of exfoliated 2D MoS_2_ is ~1.3 g h^−1^, which is one to two orders of magnitude higher than those of other methods for synthesizing MoS_2_-based or even other TMDC-based catalysts using the most ideal assumptions. Our catalyst also works well at high current densities up to 1000 mA cm^−2^ giving it a big advantage in both production rate and working current density. The 2D MoS_2_ ink also has the advantage of being able to be added to electrodes by spraying and dipping. The MoS_2_ mineral-based catalyst also has a low price of only 10 US$ m^−2^ excluding the cost of the support, almost 30 times lower than commercial Pt/C catalysts (Fig. 5b and Supplementary Table 5). Possible replacement of the Mo_2_C additive by cheaper materials would further reduce the overall cost. While the result presented here is a notable achievement, we note that a more comprehensive analysis will be the subject for future study. Taken together, these results indicate the huge potential of our high-throughput method for fabricating cheap, high-performance, and durable MoS_2_-based catalysts from minerals that are suitable for large-scale H_2_ production.

## Discussion

We have reported a high-throughput and scalable method for production of MoS_2_-based ink-type electrocatalysts, which combines scalable top-down exfoliation and simple thermal treatment. The catalysts exhibit decent performance for high-current-density HER, which is also verified by using mineral. Note that our catalyst with good high-current-density HER performance has been produced from cheap and earth abundant molybdenite mineral nature resources, giving it a huge potential for large-scale industry hydrogen production. Besides molybdenite minerals for HER reported in this work, the method could be extended to the exfoliation of other layer materials from abundant natural resources for the mass production of electrocatalysts toward different electrochemical technologies.

## Methods

### Exfoliation of 2D MoS_2_

All chemicals were used as received without further purification. The 2D MoS_2_ was exfoliated by modifying a method previously reported^28^. The bulk MoS_2_ (10 g, with an average particle size of 1–2 μm) and the Mo_2_C (20 g, with an average particle size of 44 μm) powders were mixed together and added into a rotational grinding apparatus (Retsch RM 200, Germany). As a result, the MoS_2_ was exfoliated into 2D MoS_2_ after grinding for 9 h in an ambient atmosphere. For mineral exfoliation, molybdenite concentrate (20 g, from the Sandaozhuang open-pit mine, Luoyang, China) was used in place of the commercial MoS_2_ powder.

### Thermal treatment of 2D MoS_2_ to form HC-MoS_2_/Mo_2_C electrocatalyst

First, the 2D MoS_2_ was loaded onto a Cu foam that had been cleaned by diluted HCl, water, and acetone. The exfoliated 2D MoS_2_ (600 mg) was added to a mixture of ethanol (18 mL) and water (2 mL) and shaken for 10 s to produce a suspension was dropped onto the Cu foam (1 × 1 cm^2^) with different mass loadings (2−12 mg 2D MoS_2_). Other conductive supports, Ti substrate and carbon cloth, were also examined. Second, the conductive supports loaded with 2D MoS_2_ were thermal treated. In a typical procedure, Cu foam loaded with 2D MoS_2_ was placed in a quartz boat in the center of a 1.5 in. diameter quartz tube furnace. For H_2_ treatment, the furnace was first kept at given temperature (650, 750, or 850 °C) in a mixture of Ar (25 sccm) and H_2_ (7.5 sccm) for different times (10, 30, 100, or 180 min), after which S vacancies had been formed in the 2D MoS_2_. This was followed by CH_4_ treatment, in which the H_2_-treated sample was held at an optimum temperature of 750 °C in a mixture of Ar (25 sccm), H_2_ (2.5 sccm) and CH_4_ (2.5 sccm) for different times (10, 30, 60 or 180 min) in order to partially convert the MoS_2_ into Mo_2_C nanocrystals. The final optimized sample is denoted HC-MoS_2_/Mo_2_C. The Pt/C electrocatalysts loaded on high-surface-area Cu foams were treated under the same optimized conditions.

### Materials characterization

The surface morphology of the HC-MoS_2_/Mo_2_C samples was characterized by SEM (5 kV, Hitachi SU8010, Japan). The thickness of the 2D MoS_2_ flakes was measured by AFM (Bruker Dimension Icon, Germany). TEM and HRTEM were carried out by using an electron acceleration voltage of 300 kV (FEI Tecnai F30, USA). Structural and chemical analyses of the samples were performed by powder XRD (Cu Kα radiation, λ = 0.15418 nm, Bruker D8 Advance, Germany), while Raman spectra were collected using 532 nm laser as the excitation light with a beam size of ~1 μm (Horiba LabRAB HR800, Japan).

### Electrochemical measurements

A standard three-electrode electrolyzer with H_2_SO_4_ (0.5 M) or KOH (1.0 M) was used in all tests, with a saturated calomel electrode (SCE) and a graphite rod as the reference and counter electrodes, respectively. Pt counter electrode is used for taking videos. The scan rates were 5 mV s^−1^ for the linear sweep voltammetry tests and the scan rates for the cyclic voltammetry tests have been noted in the captions of the related figures. For fair comparison, a 85% *iR* correction was taken. Electrochemical active surface areas (ECSA) were obtained by measuring electrochemical double layer capacitance (*C*_dl_) of catalysts. Faradaic efficiencies were defined as the ratio of H_2_ amount collected in experiment to the amount in theory, where H_2_ was collected by water drainage method. Stability tests were performed by chronoamperometry measurements method.

## Supplementary information

Supplementary infomation

Peer Review File

Summlementary Movie 1

Summlementary Movie 2

## Data Availability

Source data are provided with this paper.

## Source data

Source Data
