# Supplementary material for: High-throughput production of cheap mineral-based two-dimensional electrocatalysts for high-current-density hydrogen evolution
Source: Nat Commun. 2020 Jul 24;11:3724. doi: 10.1038/s41467-020-17121-8 (PMC7381654; doi:10.1038/s41467-020-17121-8)
Supplement: Supplementary file 1 — Supplementary infomation [file 41467_2020_17121_MOESM1_ESM.pdf]

Supplementary Information for ***Nature Communications***

High-Throughput Production of Cheap Mineral-Based Two-dimensional Electrocatalysts for High-Current-Density Hydrogen Evolution

Zhang *et al.*

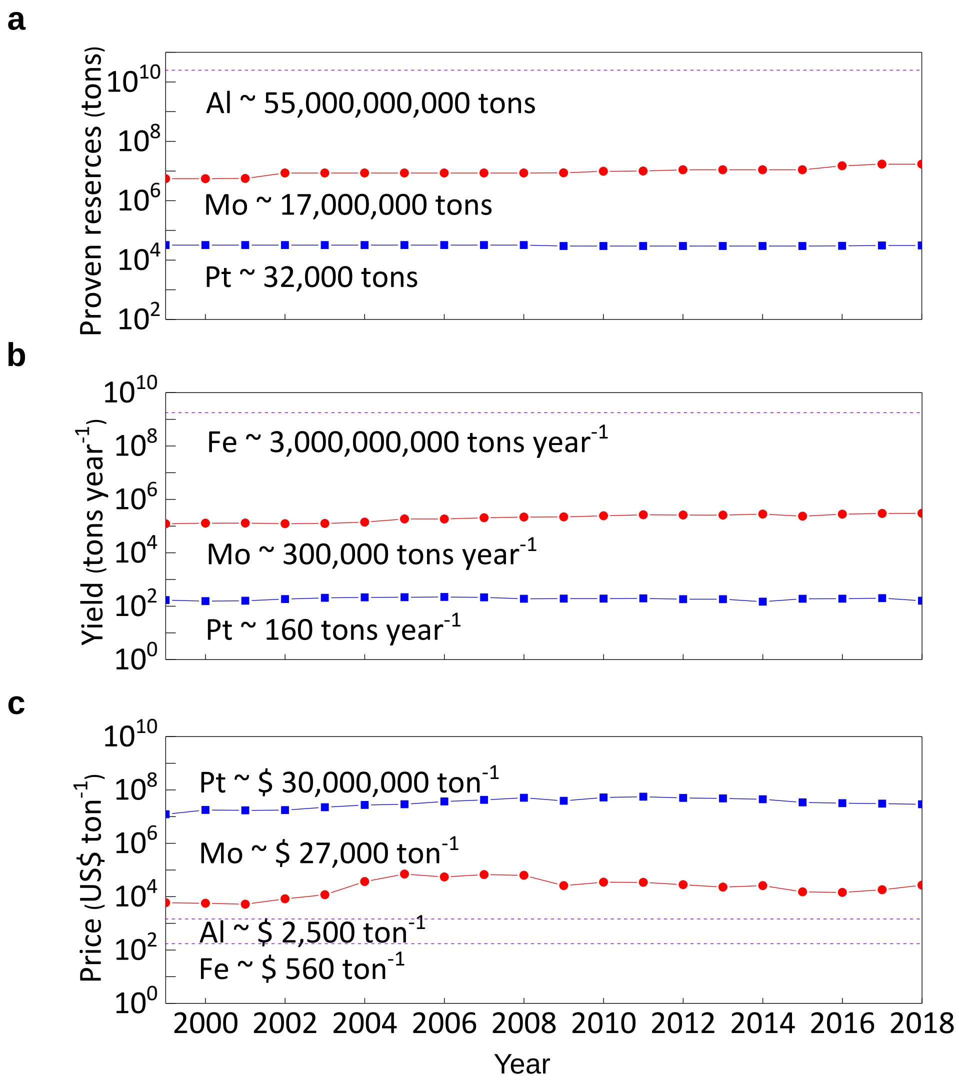

Supplementary Figure 1. Commodity statistical data of Pt, Mo, Al, and Fe metals. a Proven reserves, **b** annual yield, and **c** commodity price of these metals in the past 20 years, according to the United States Geological Survey (USGS) annual publications.[1] The Earth proven reserve of Pt is only about 32,000 tons, which is six orders of magnitude less than that of the most abundant element, *i.e.*, Al. These identified reserves only increased by 900 tons (2.8%) in the last 20 years, also suggesting the scarcity of Pt element. The global yield of Pt is only about 160 tons per year, which is 10 million time less than that of the most produced metal, Fe (Pig iron and raw steel). Combined with its scarcity and low yield, Pt keeps a very high price of about 3×10^7 US$ ton^{-1}, while Mo (2.7×10^4 US$ ton^{-1}) is much cheaper than Pt.

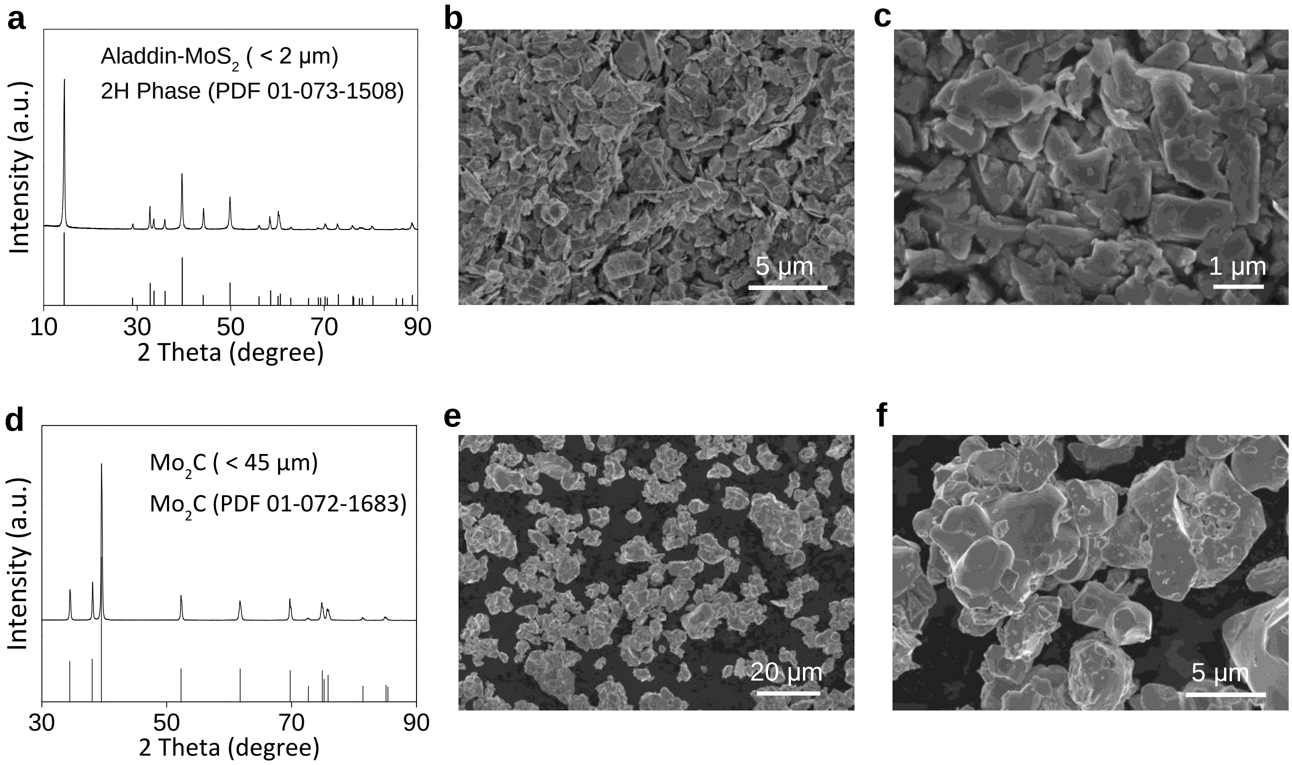

Supplementary Figure 2. Characterization of the raw materials. a-c The high-purity bulk MoS_2 and **d-f** the Mo_2C force intermediates used in the intermediate-assisted grinding exfoliation (iMAGE) process. **a**,**d** XRD patterns, **b**,**d** low and **c**,**f** high magnification SEM images.

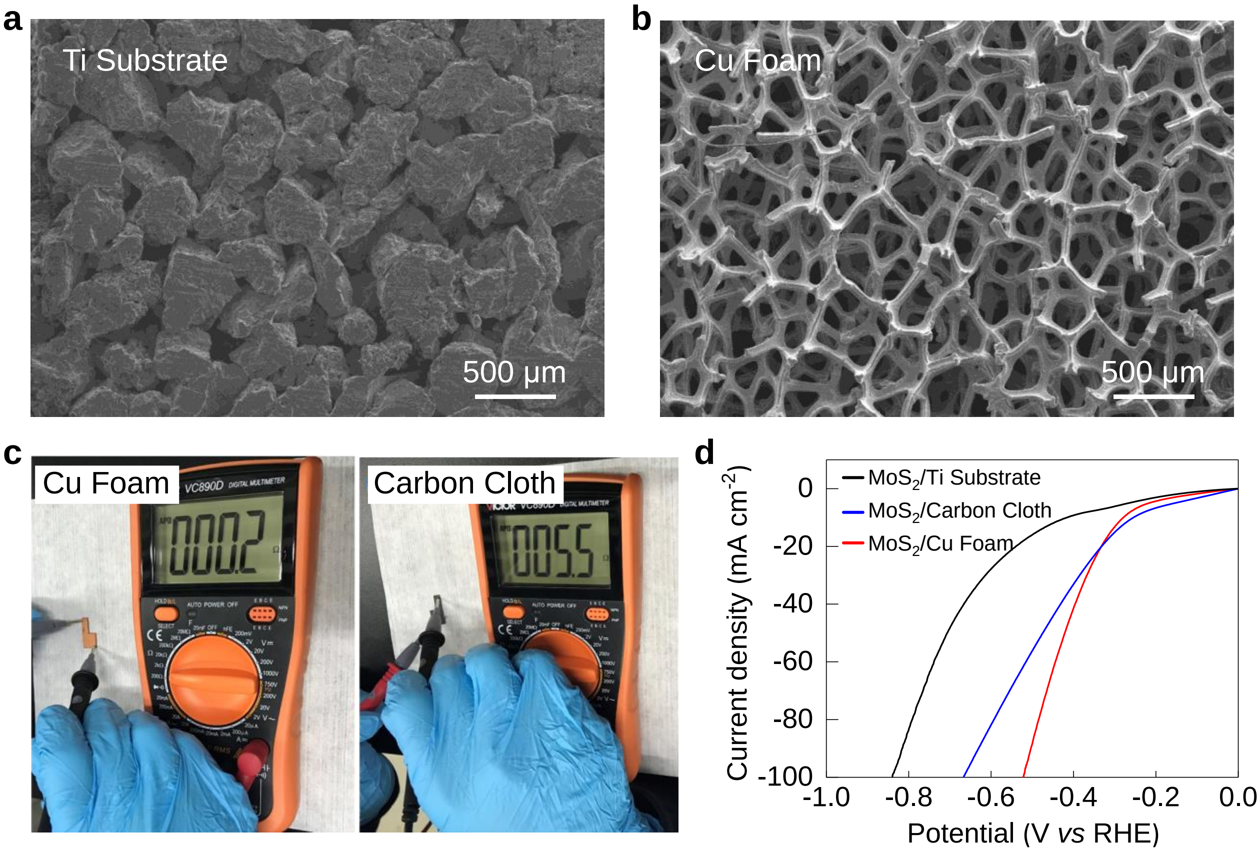

Supplementary Figure 3. 2D MoS_2 electrocatalysts prepared on different supports by the method in Figure 1a in the main text. **a**, **b** SEM images of the **a** Ti substrate and **b** Cu foam supports. **c** Electrical resistance of Cu foam and carbon cloth with same size. **d** HER polarization curves of the 2D MoS_2 catalyst (4 mg cm^{-2}) on three different supports. The results show that 2D MoS_2 loaded on all three conductive supports show certain HER activity, and the performance is related to the porosity and electrical conductivity of the supports.

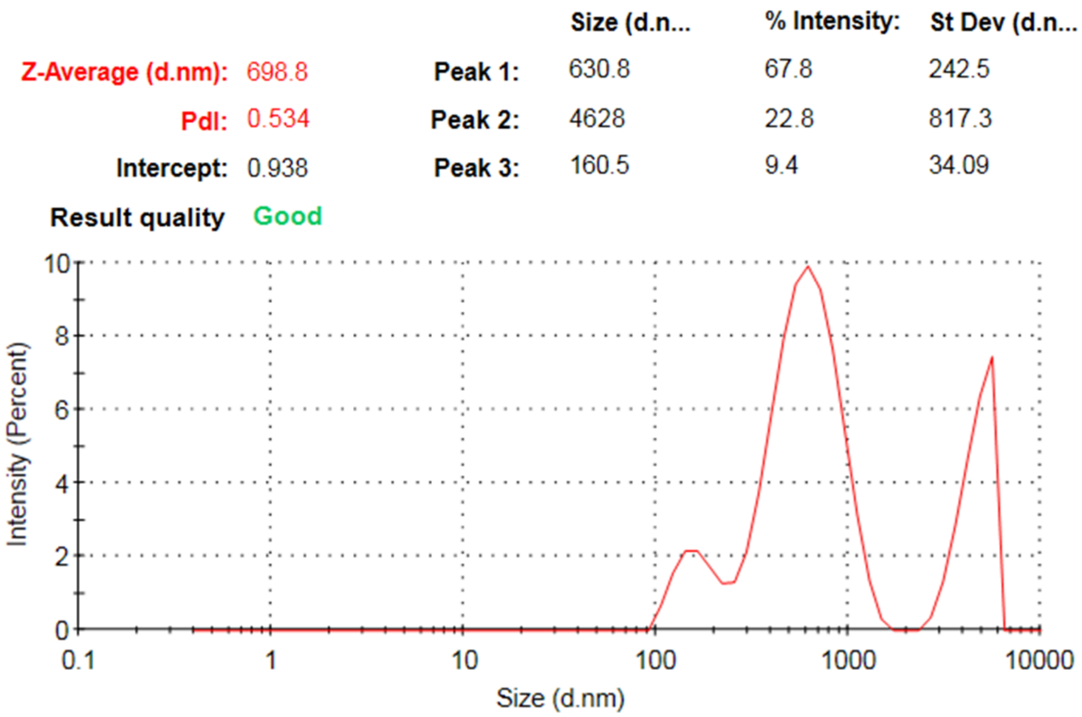

Supplementary Figure 4. Dynamic light scattering (DLS) measurements of the exfoliated 2D MoS_2. The results show that the average flake size is about 700 nm, consistent with the AFM and TEM results.

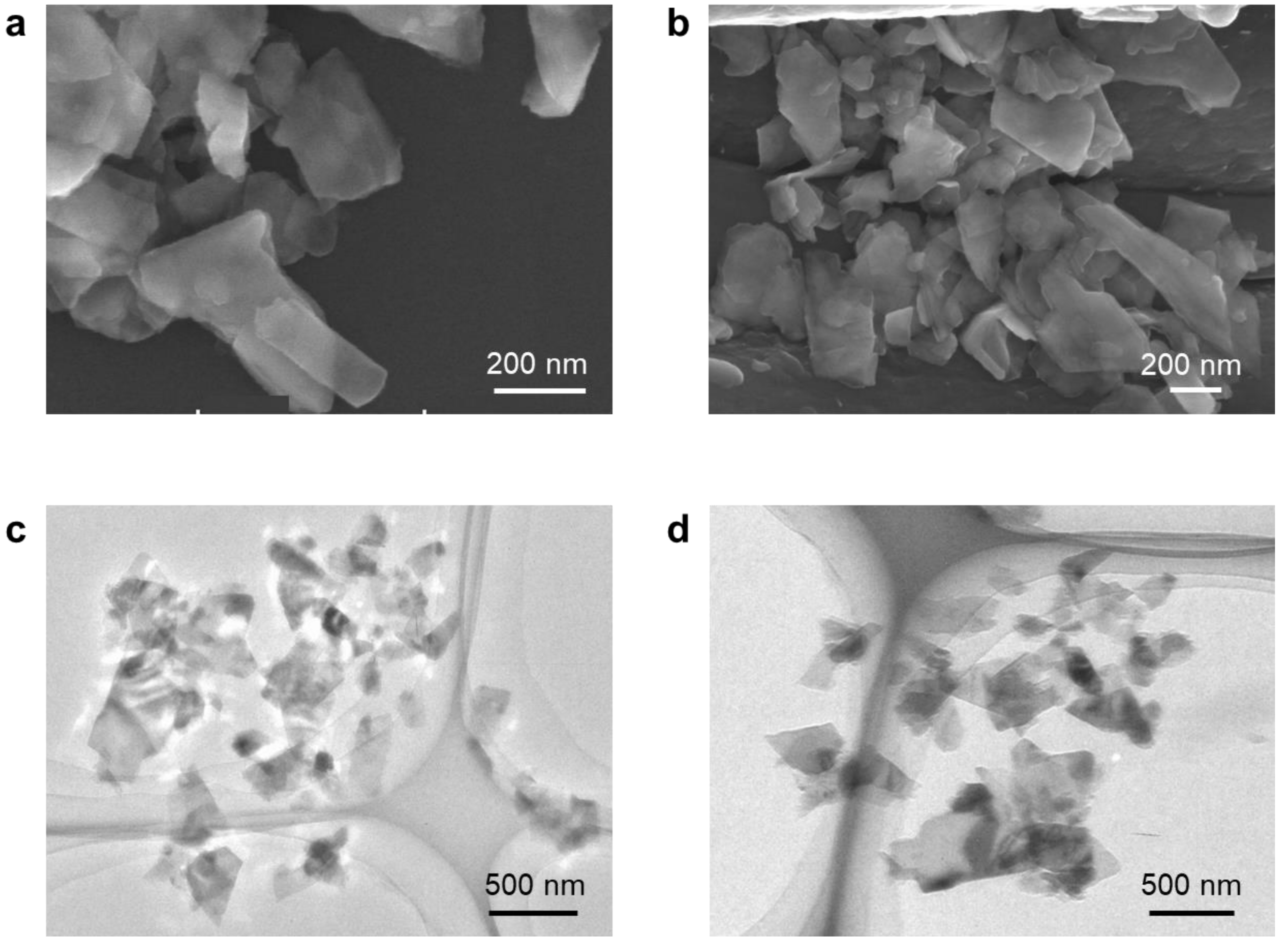

Supplementary Figure 5. Morphology of the iMAGE method **exfoliated 2D MoS_2 flakes. a**,**b** SEM and **c**,**d** TEM images.

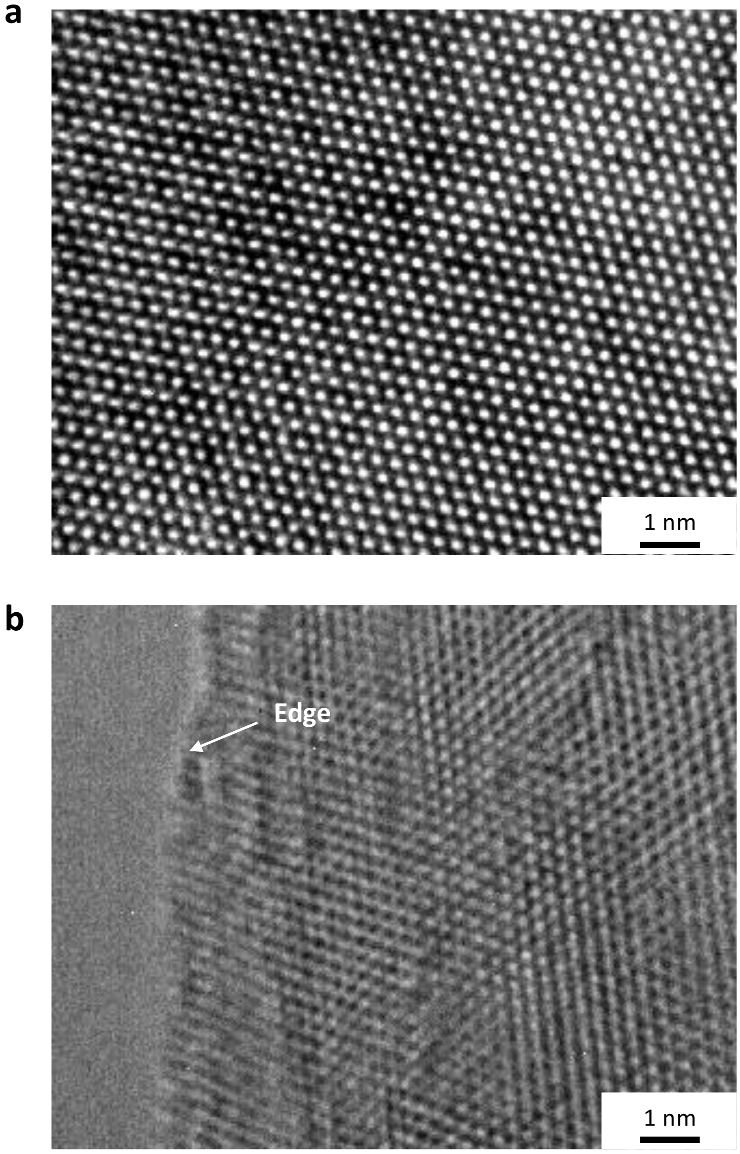

Supplementary Figure 6. HRTEM images of the exfoliated 2D MoS_2. a the basal plane and **b** the edge of a 2D MoS_2 flake.

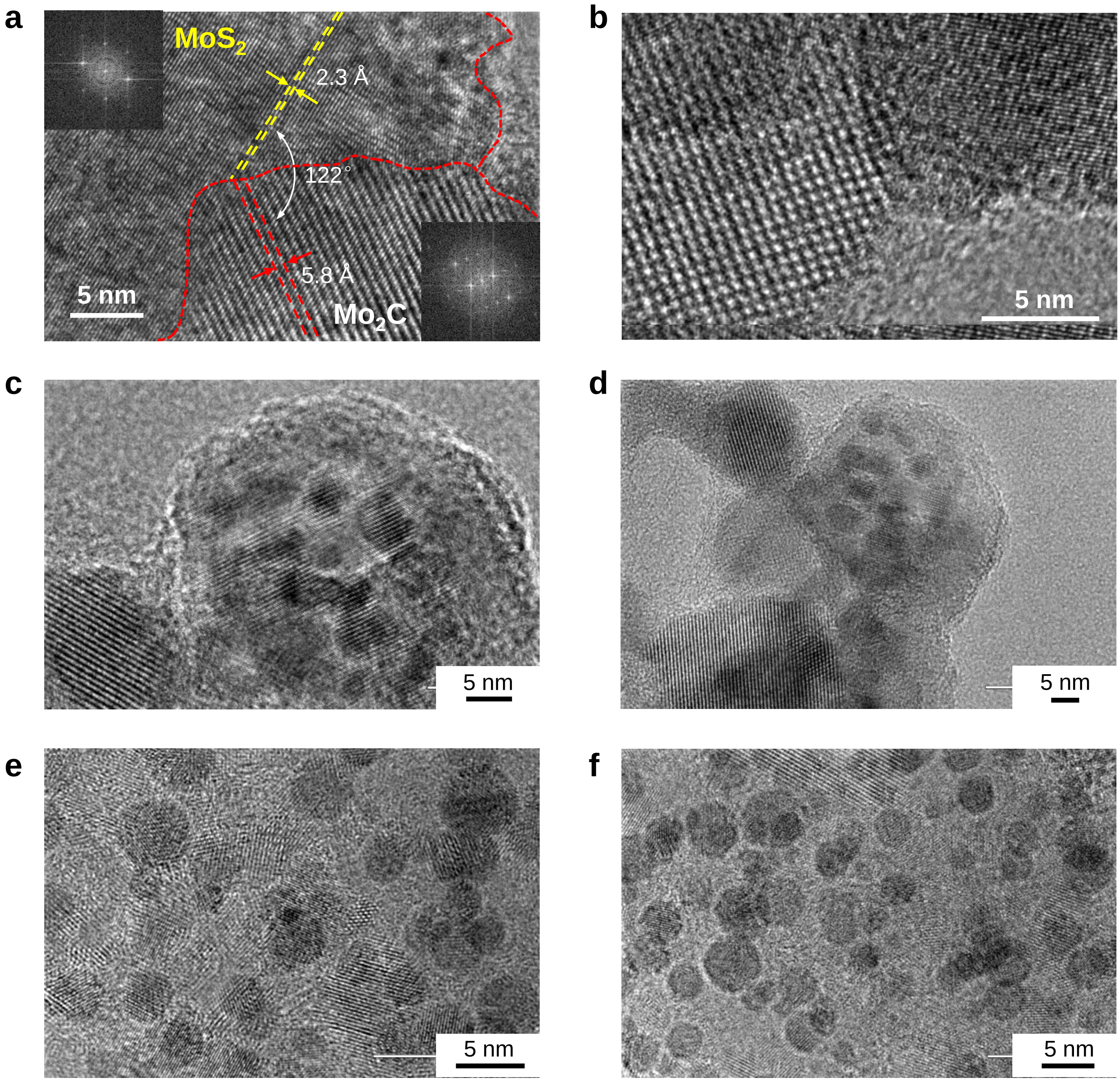

Supplementary Figure 7. Microstructural of HC-MoS_2/Mo_2C electrocatalysts. a-f HRTEM images of the HC-MoS_2/Mo_2C electrocatalyst, showing the formation of many nanosized MoS_2/Mo_2C lateral heterostructures. The Mo_2C nanocrystals are decorated the basal planes and edges of 2D MoS_2 flakes. In Figure S7a, the *d*-spacing of 2.3 Å is corresponding to that of MoS_2 (103) facet, and the d-spacing of 5.8 Å is in agreement with Mo_2C lattice constant in the b direction.[2] At the boundary of Mo_2C and MoS_2, the intersection angle between them is 122°, a value close to the 120° due to the three-fold symmetry of the MoS_2. These results indicated that the matrix structure of MoS_2 is important for obtaining MoS_2/Mo_2C heterostructures.

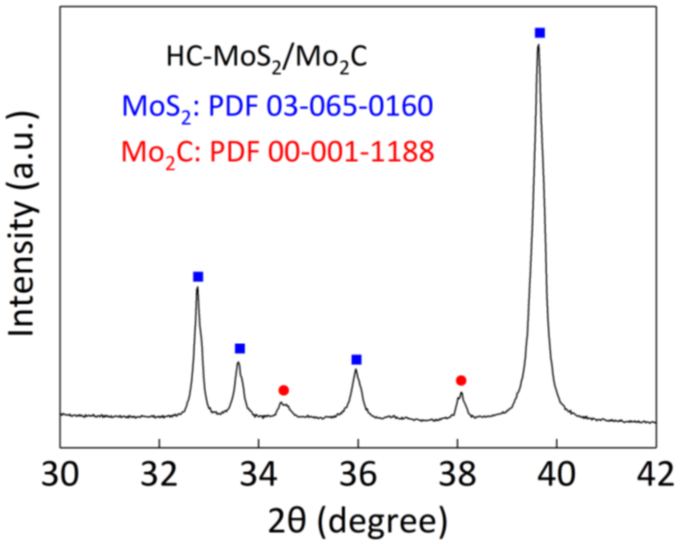

Supplementary Figure 8. Fine structure of the XRD pattern of the HC-MoS_2/Mo_2C catalyst. The result shows that the HC-MoS_2/Mo_2C is composed of MoS_2 and Mo_2C.

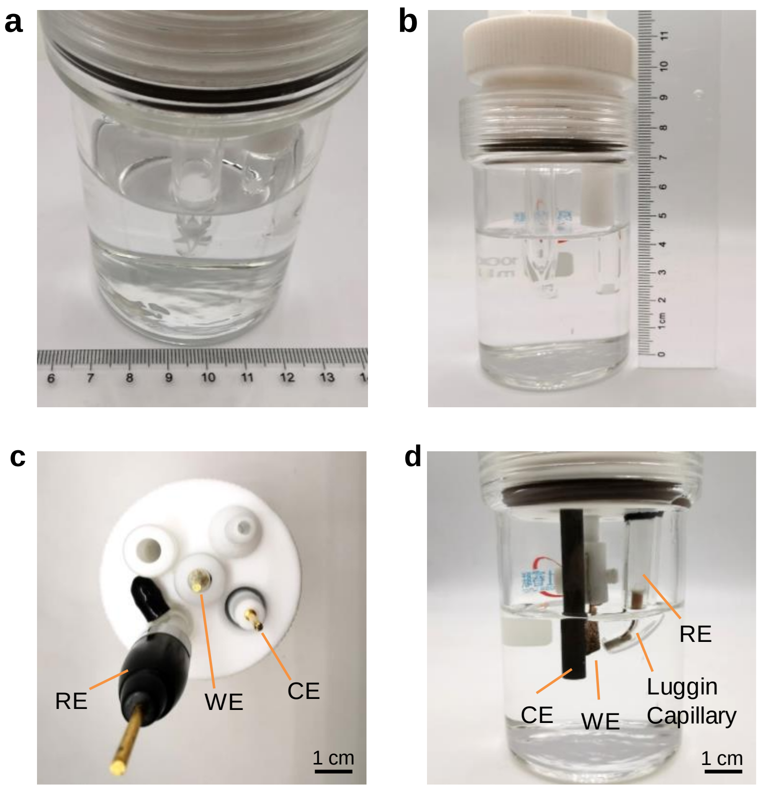

Supplementary Figure 9. Photos of the electrolysis cell for performing electrochemical tests. a,b Photos showing the size of the electrolysis cell. **c,d** Photos showing the positions and distances between working, counter, and reference electrodes. These photos show that the cell and test parameters were kept identical in each test to make the electrochemical test reliable.

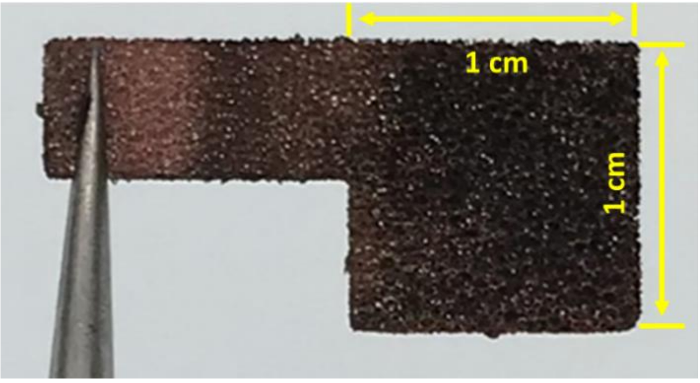

Supplementary Figure 10. A photo of a HC-MoS_2/Mo_2C working electrode. It shows an apparent surface area of 1 cm^2.

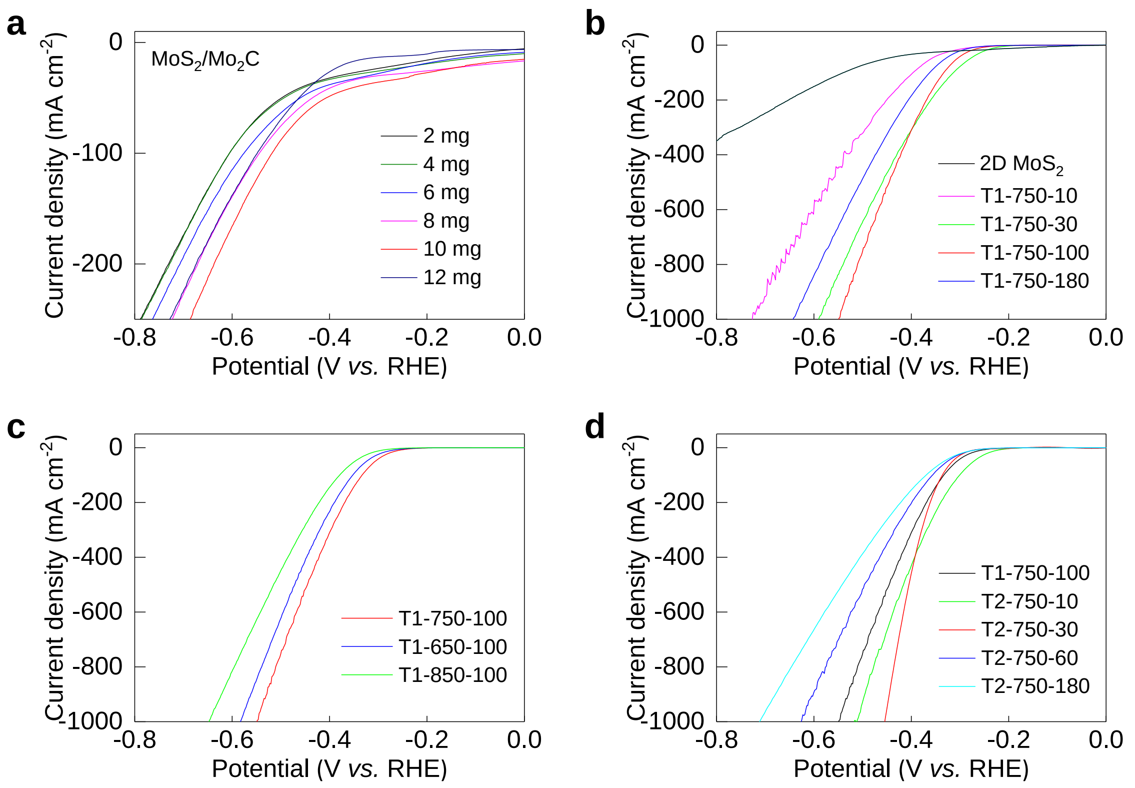

Supplementary Figure 11. Optimization of the HER performance of the MoS_2-based catalysts. The tuned parameters include **a** loading amounts of 2D MoS_2 on Cu foam, **b** time in the hydrogen treatment process, **c** temperature in the hydrogen treatment process, and **d** time in the carbonization treatment process. In a typical procedure, Cu foam loaded with 2D MoS_2 (2-12 mg cm^{-2}) was placed in a quartz boat in the center of a 1.5 in. diameter quartz tube furnace. For H_2 treatment (denoted as T1), the furnace was first kept at given temperature (650, 750, or 850 °C) in a mixture of Ar (25 sccm) and H_2 (7.5 sccm) for different times (10, 30, 100, or 180 min), after which S vacancies had been formed in the 2D MoS_2. This was followed by CH_4 treatment (denoted as T2), in which the H_2-treated sample was held at an optimum temperature of 750 °C in a mixture of Ar (25 sccm), H_2 (2.5 sccm) and CH_4 (2.5 sccm) for different times (10, 30, 60 or 180 min) in order to partially convert the MoS_2 into Mo_2C nanocrystals. All these samples are denoted in the form of "T1/2-temperature-time". The first-step optimized H_2 treated sample (T1-750-100) is denoted as H-MoS_2, and the final optimized sample (T2-750-30) is denoted as HC-MoS_2/Mo_2C. All tests were done in 0.5 M H_2SO_4.

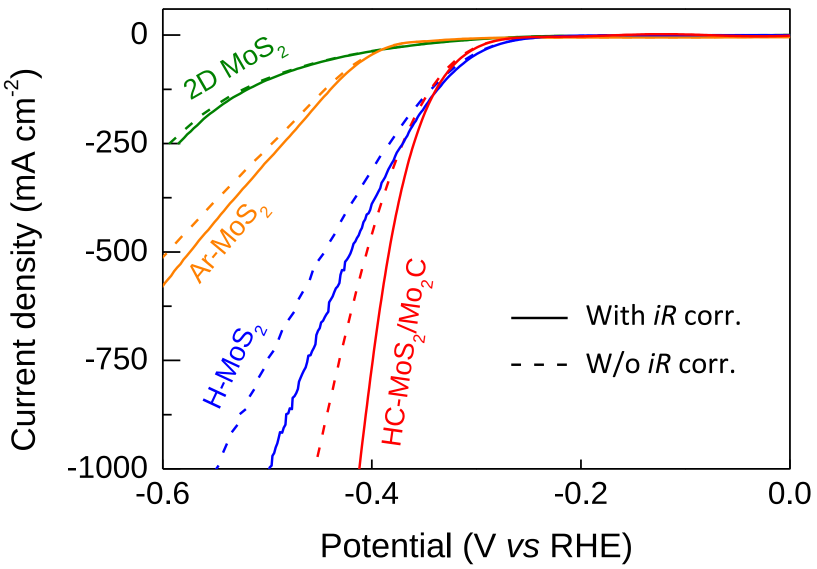

Supplementary Figure 12. Optimization of HER performance of MoS_2-based electrocatalysts. Polarization curves of different MoS_2-based samples **a** without *iR* correction and **b** with *iR* correction. The catalysts of 2D MoS_2 with only Ar and Ar/H_2 treatments are denoted as Ar-MoS_2 and H-MoS_2. All tests were done in 0.5 M H_2SO_4.

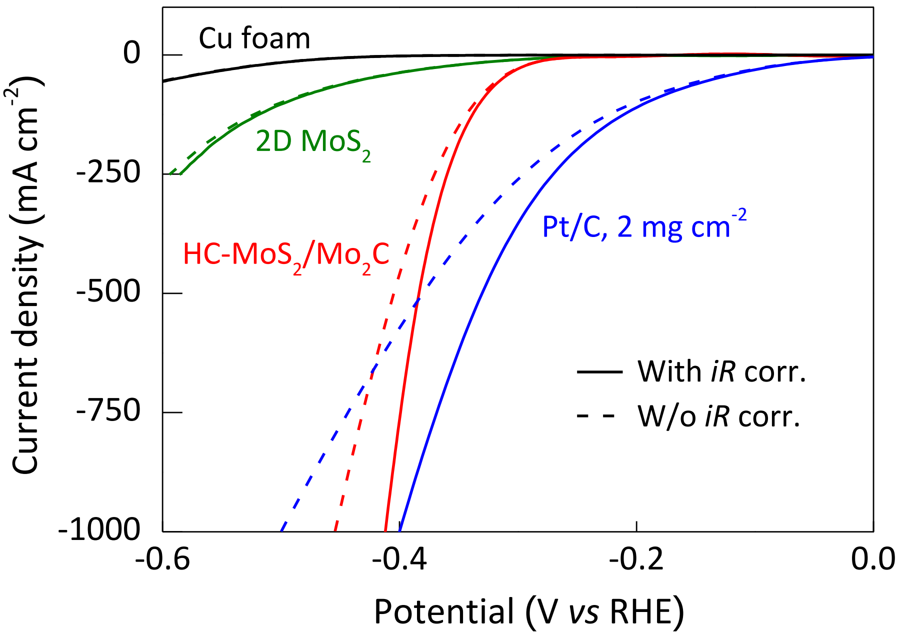

Supplementary Figure 13. Polarization curves. Samples include HC-MoS_2/Mo_2C, 2D MoS_2, Pt/C loaded on Cu foam (2 mg cm^{-2}), and bare Cu foam. The tests were done in 0.5 M H_2SO_4 electrolytes, where the data with *iR* correction were shown in Figure 3a in the manuscript.

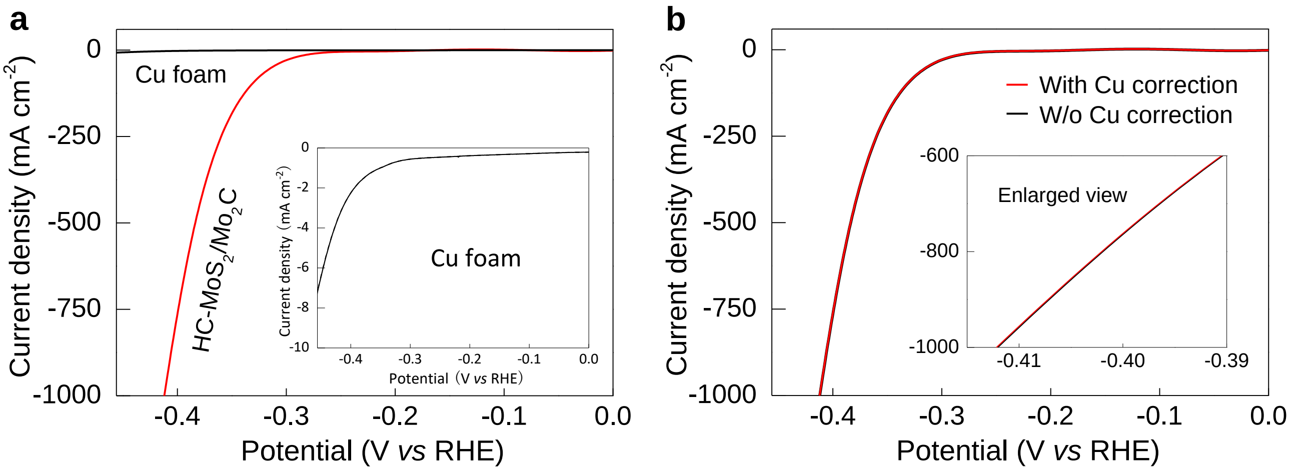

Supplementary Figure 14. The effect of Cu foam on catalyst performance. a Polarization curves of the HC-MoS_2/Mo_2C on Cu foam and the bare Cu foam. The inset is the enlarged view of the Cu foam. The results showed that the performance of Cu foam in the potential window of HC-MoS_2/Mo_2C is negligible. **b** Polarization curves of HC-MoS_2/Mo_2C before and after being corrected by Cu signals. The inset is the enlarged view at high current densities. The solid lines and dotted lines denote data with and without *iR* corrections, respectively. All tests were done in 0.5M H_2SO_4. These results showed that the effect of Cu foam on current densities of catalysts are negligible. All data are *iR* corrected.

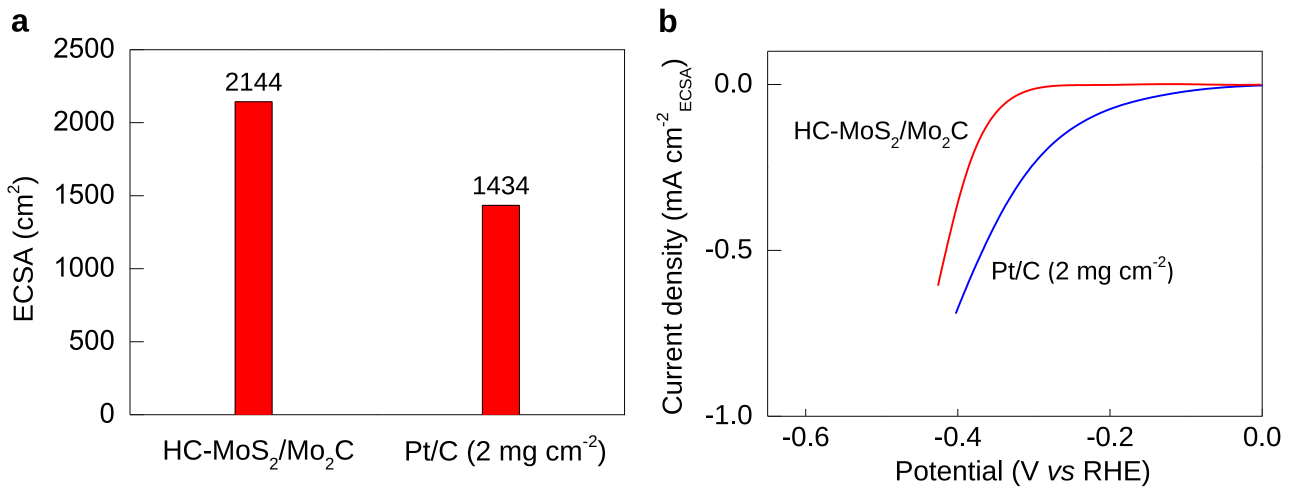

Supplementary Figure 15. Electrochemically active surface areas (ECSA) normalized current densities. Samples include HC-MoS_2/Mo_2C and the Pt/C loaded on a high-surface-area Cu foam (2 mg cm^{-2}) and are tested in 0.5 M H_2SO_4 solutions. **a** ECSA values and **b** ECSA-normalized polarization curves of the HC-MoS_2/Mo_2C and the 2 mg cm^{-2} Pt/C with *iR* correction. The ECSA were calculated from their double layer capacitance (C_{dl}) as shown in Figure S18,[3,4] which were 2144 cm^2 for the HC-MoS_2/Mo_2C and 1434 cm^2 for the 2 mg cm^{-2} Pt/C. These results showed that the Pt catalyst had higher intrinsic activity for HER than the HC-MoS_2/Mo_2C catalyst.

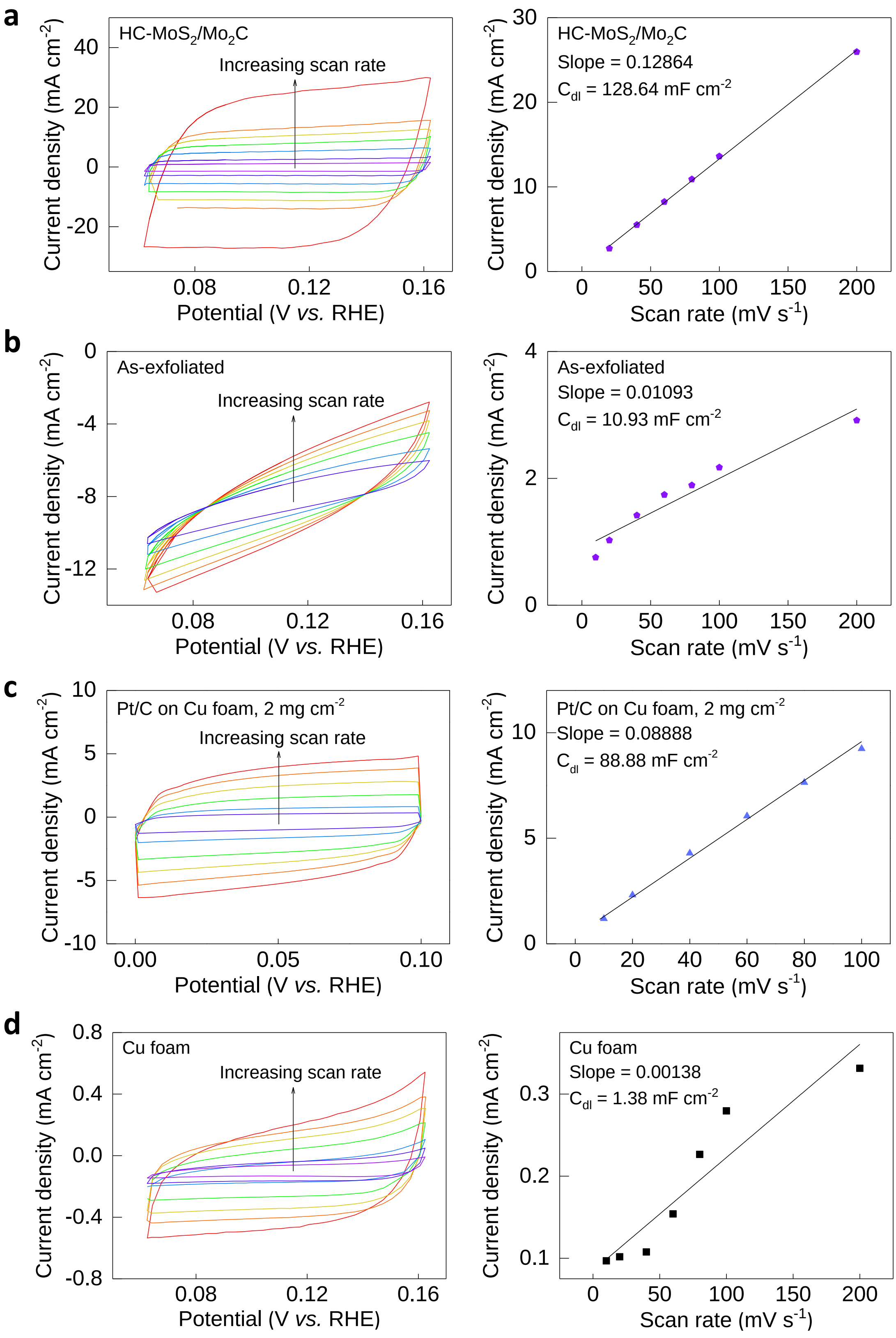

Supplementary Figure 16. CV curves at different scan rates and the calculated C_{dl}. **a** HC-MoS_2/Mo_2C, **b** As exfoliated 2D MoS_2, **c** Pt/C loaded on high-surface-area Cu foam with a loading mass of 2 mg cm^{-2}, and **d** bare Cu foam in 0.5 M H_2SO_4.

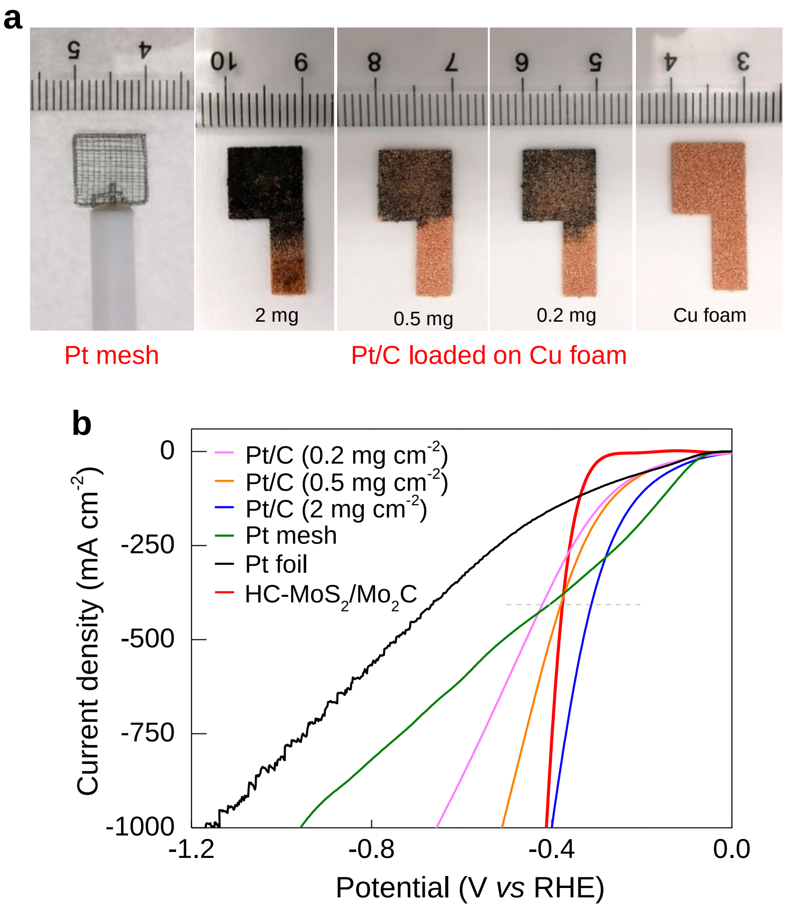

Supplementary Figure 17. Morphology engineering of Pt-based electrocatalysts toward better high-current-density performance. a Optical image of a series of Pt working electrode including Pt foil, Pt mesh, Pt/C (0.2, 0.5, and 2 mg cm^{-2}) loaded on high-surface-area Cu foams, with an apparent surface area of 1 cm^2. **b** Polarization curves of HC-MoS_2/Mo_2C and these Pt catalysts tested in 0.5 M H_2SO_4 with *iR* correction. The results showed that Pt/C on Cu foam with a high electrochemical surface area (Figure S16c) exhibited the best performance among these Pt-based electrocatalysts, and HC-MoS_2/Mo_2C exhibited comparable performance with the best Pt catalyst.

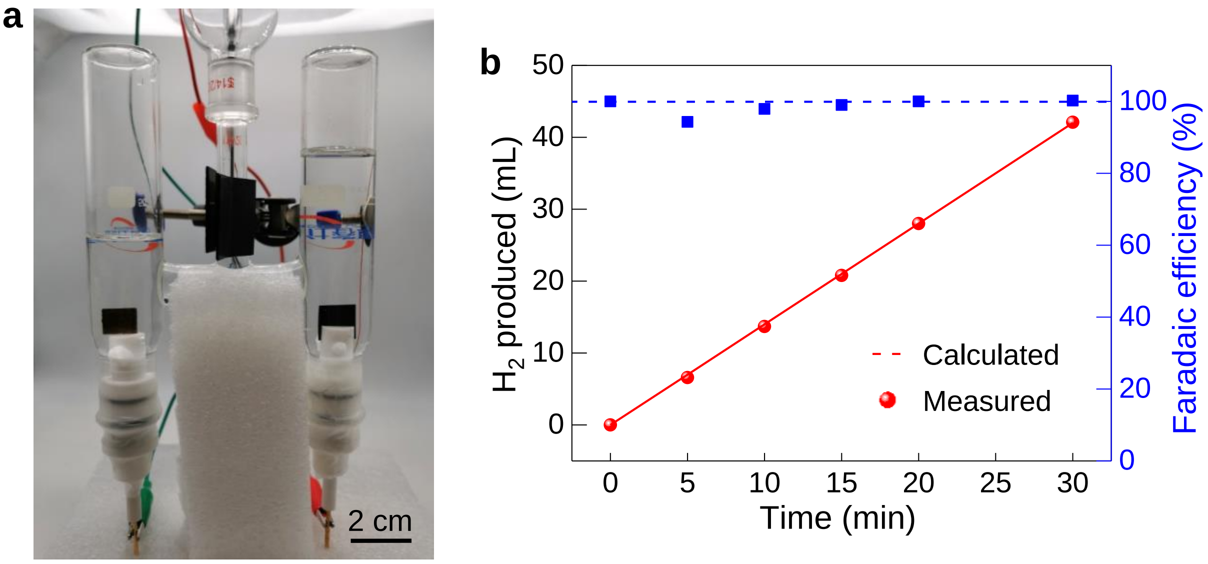

Supplementary Figure 18. Measurements of the Faradaic efficiency of the HC-MoS_2/Mo_2C sample. **a** A photo of the setup for testing Faradaic efficiency and **b** the volume of hydrogen with different durations during HER. The tests were done at 200 mA cm^{-2}. The results showed that the HC-MoS_2/Mo_2C catalyst exhibited a ~100% Faradaic efficiency for HER, and the side reaction from Cu foam would be negligible.

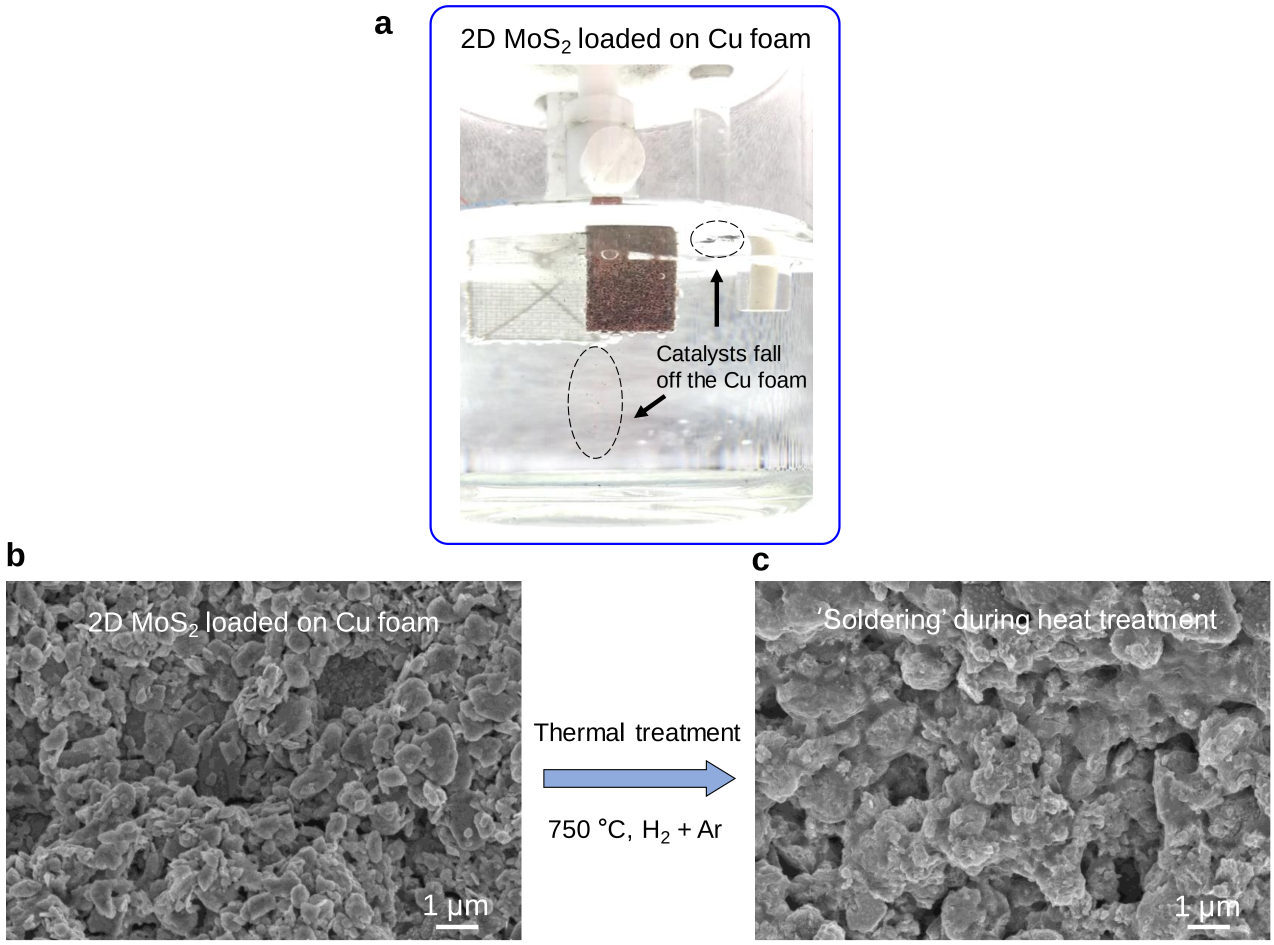

Supplementary Figure 19. Soldering phenomenon of 2D MoS_2 catalyst on Cu foam during thermal treatment. a Optical image and **b** SEM image for the catalyst without thermal treatment after loading on the Cu foam. **c** SEM image for the catalyst after thermal treatment. During HER tests, we observed that the catalyst easily falls off the Cu foam for **b**, while it is firmly attached on the Cu foam for **c**. More details are shown in Supplementary Movies 1 and 2.

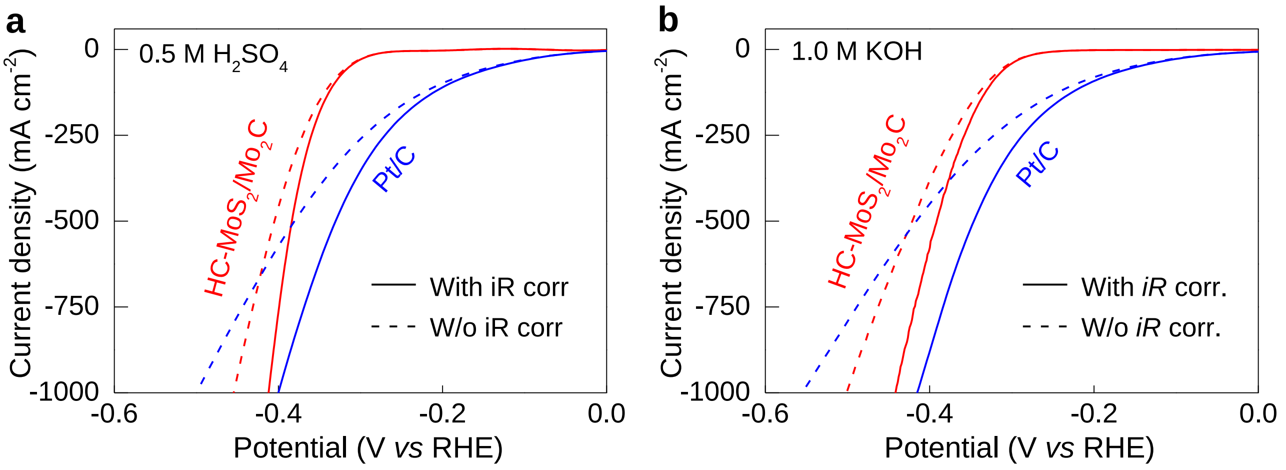

Supplementary Figure 20. The pH-universal HER performance of the HC-MoS_2/Mo_2C catalysts. Polarization curves of HC-MoS_2/Mo_2C and 2.0 mg cm^{-2} Pt/C in 0.5 M H_2SO_4 and 1.0 M KOH **a** without *iR* correction and **b** with *iR* correction. The results showed that HC-MoS_2/Mo_2C had good performance in both acidic and alkaline media.

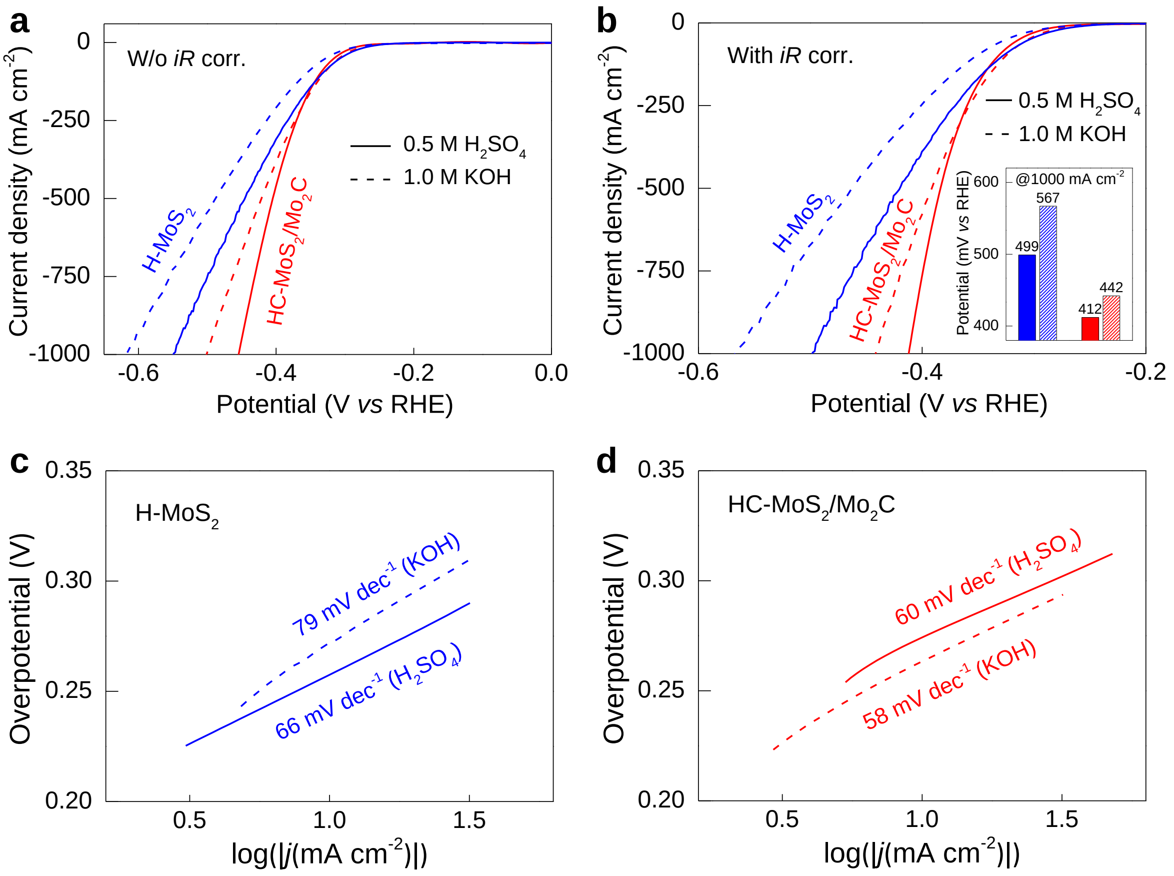

Supplementary Figure 21. The pH-universal HER performance of the HC-MoS_2/Mo_2C in 0.5 M H_2SO_4 and 1.0 M KOH. Polarization curves of H-MoS_2 and HC-MoS_2/Mo_2C **a** without and **b** with *iR* correction. The inset of **b** is their corresponding overpotentials at 10 mA cm^{-2}. Tafel plots of **c** H-MoS_2 and **d** HC-MoS_2/Mo_2C in different electrolytes. The results showed that HC-MoS_2/Mo_2C is pH-universal and Mo_2C may play important role in the performance improvement of the HC-MoS_2/Mo_2C catalyst in alkaline media.

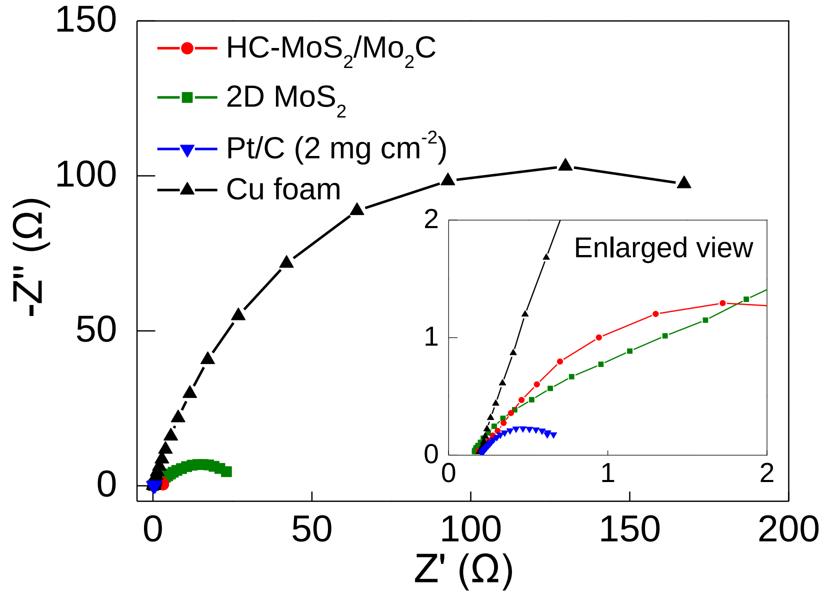

Supplementary Figure 22. EIS curves. Samples including HC-MoS_2/Mo_2C, exfoliated 2D MoS_2, 2 mg cm^{-2} Pt/C, and Cu foam were tested in 0.5 M H_2SO_4 solutions. The results show that we could reduce R_s for all catalysts to similar small values.

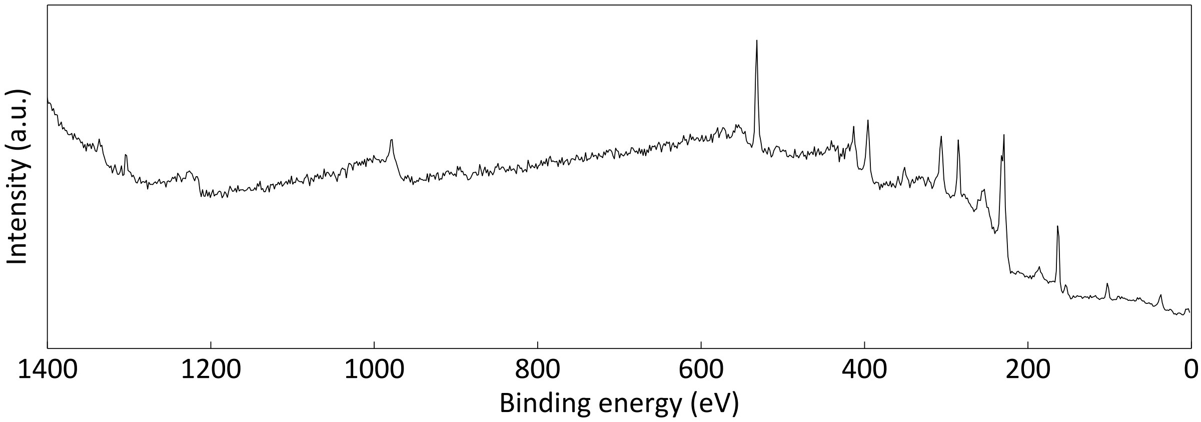

O	S	Mo	Si	Mg	Ca	Al	Fe	W	Na
39.0%	24.4%	13.7%	10.7%	4.4%	3.0%	1.4%	1.4%	1.2%	0.9%

Supplementary Figure 23. XPS results of molybdenite concentrate. The percentages of different elements are in a unit of at%. These results show that large amounts of impurities such as silicate are in the surface of the concentrate.

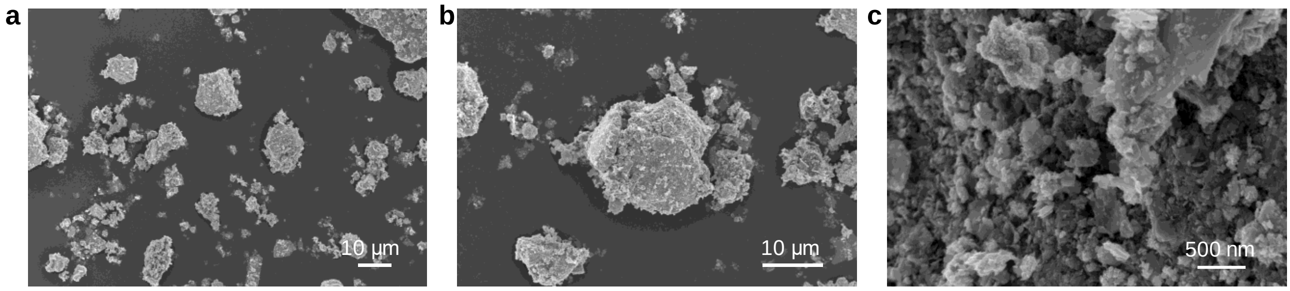

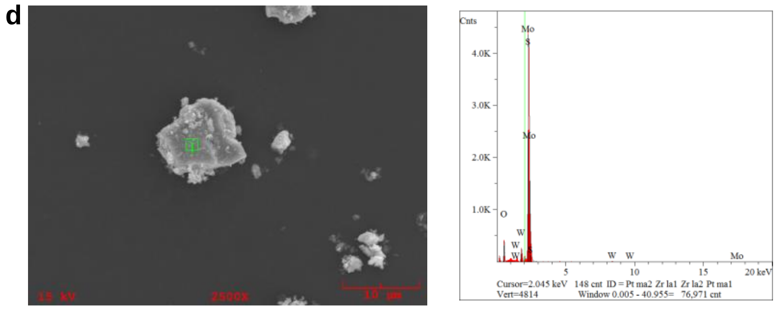

Elt.	Line	Intensity (c/s)	Atomic %	Conc	Units	MDL 3-sig	
O	Ka	116.18	42.986	16.00	wt.%	0.442	
S	Ka	928.58	30.513	22.76	wt.%	0.141	
Mo	La	705.76	25.473	56.85	wt.%	0.464	
W	Ma	83.28	1.028	4.40	wt.%	0.300	
			100.000	100.00	wt.%		Total

Supplementary Figure 24. Morphology and element content of the molybdenite concentrate. a-c SEM images and **d** EDS analysis of molybdenite concentrate. The results show that besides layered MoS_2, there are lots of impurities in the bulk of molybdenite concentrate.

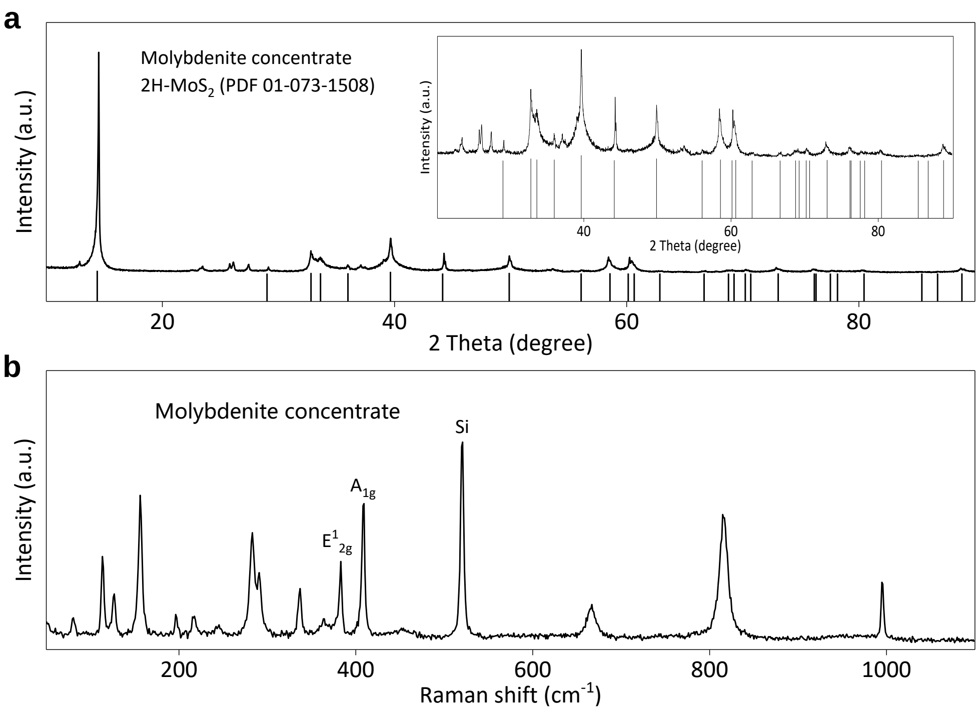

Supplementary Figure 25. Characterization of molybdenite concentrate. a XRD and **b** Raman spectrum of molybdenite concentrate. The results show it is mainly composed of 2H-phase MoS_2 (XRD result) and some molybdenum oxides (Raman result).

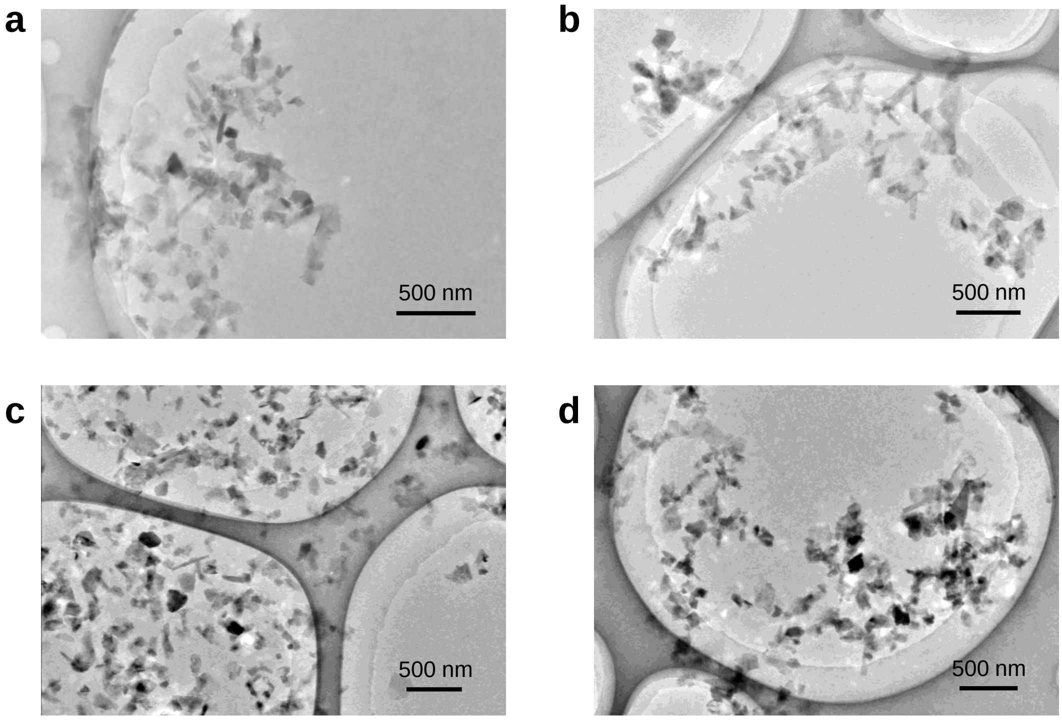

Supplementary Figure 26. TEM images of the iMAGE exfoliated 2D MoS_2 from molybdenite concentrate. a-d TEM images showing several samples of 2D MoS_2 from molybdenite concentrate.

a

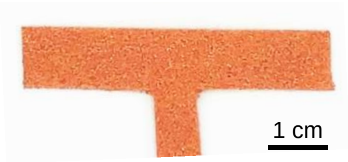

b

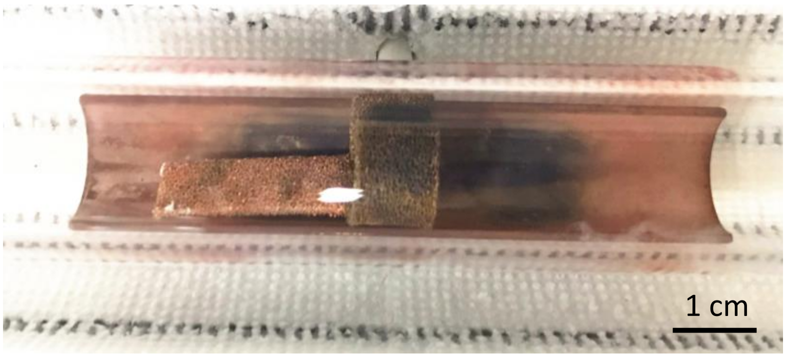

Supplementary Figure 27. Optical images of the large Cu foam sample. It has a size of 1 cm × 5 cm and the following thermal treatment.

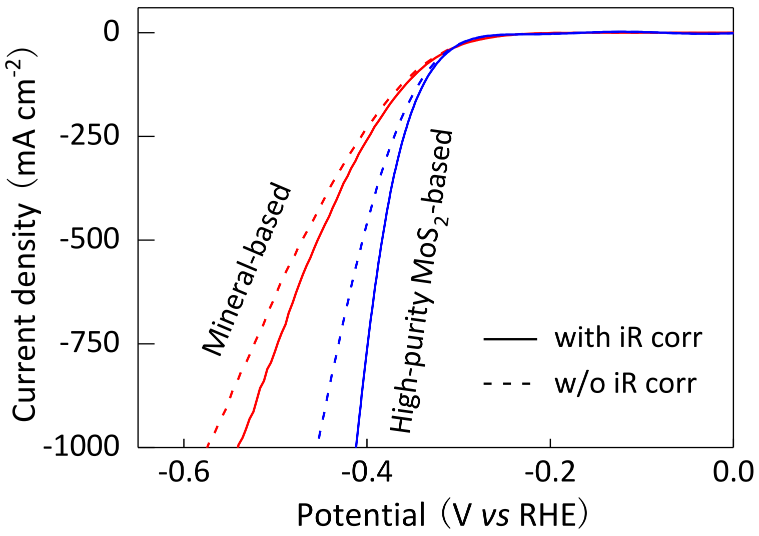

Supplementary Figure 28. HER performance. The results show the difference between the cheap MoS_2 mineral-based catalyst (molybdenite concentrate) and the high-purity MoS_2-based catalyst.

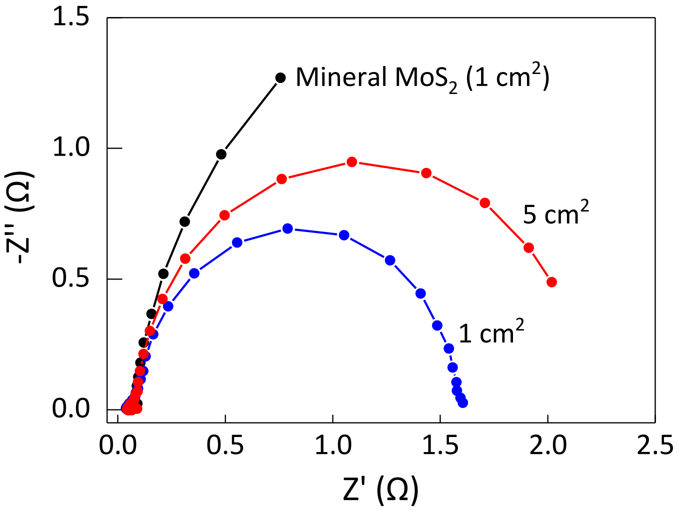

Supplementary Figure 29. EIS curves. MoS_2 mineral-based electrodes have areas of 1 cm^2 and 5 cm^2. The results showed that compared with as-received mineral, MoS_2 mineral-based electrocatalysts including 1cm^2 and 5cm^2 samples showed small charge transfer resistances for HER.

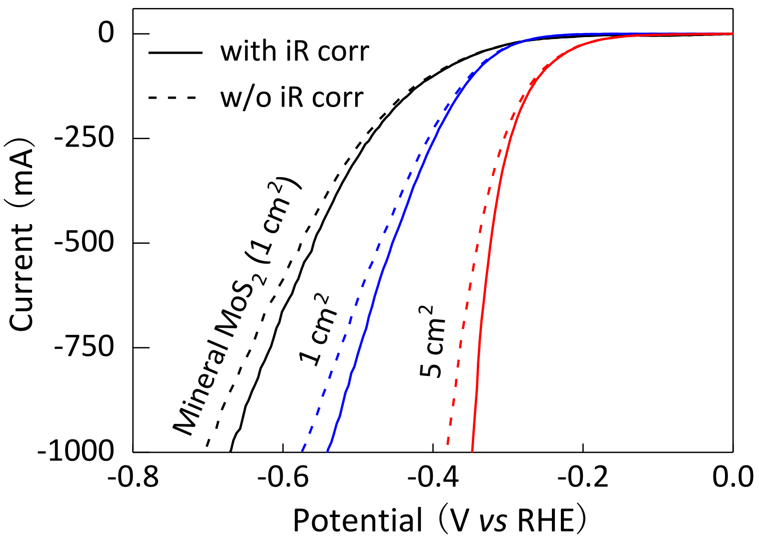

Supplementary Figure 30. Polarization curves of the different MoS_2-mineral-based catalysts. All the samples were tested in 0.5 M H_2SO_4 solutions. Curves both with and without *iR* corrections are provided for each sample.

Supplementary Table 1. A comparison of the highest current density and production rate of catalysts in this work with previously reported data.

Synthesis method	Catalysts	Highest current density (mA cm^{-2})	Production rate (mg h^{-1})	Notes	Ref
Top-down grinding exfoliation (iMAGE)	HC-MoS_2/Mo_2C	1,000	1,250	N/A	This work
Tip sonication exfoliation	Exfoliated MoS_2	200	9.5	Assumption of 100% exfoliation	5
Li intercalation exfoliation	WS_2	30	6.3	Assumption of 100% exfoliation	6
Li intercalation exfoliation	1T MoS_2	40	4.2	Assumption of 100% exfoliation	7
Li intercalation exfoliation	n-BuLi treated MoS_2	200	4.9	Assumption of 100% exfoliation	8
Gas-solid reaction	MoS_2	8	0.02	Given in the ref	9
Gas-solid reaction	$MoSe_2$	8	0.03	Given in the ref	9
Gas-solid reaction	MoS_2	200	1.6	Given in the ref	10
Gas-solid reaction	2H $Nb_{1.35}S_2$	5000	0.1	Given in the ref	11
Solvothermal synthesis	MoS_2	12	56	Assumption of 100% conversion	12
Solvothermal synthesis	MoS_2/Mo_2C	20	0.2	Given in the ref	13

Synthesis method	Catalysts	Highest current density (mA cm^{-2})	Production rate (mg h^{-1})	Notes	Ref
Solvothermal synthesis	MoS_2	30	62	Assumption of 100% conversion	14
Solvothermal synthesis	C@MoS_2	35	1.1	Assumption of 100% conversion	15
Solvothermal synthesis	MoS_2	40	0.05	Assumption of 100% conversion	16
Solvothermal synthesis	MoS_2/N-RGO	50	8.8	Assumption of 100% conversion	17
Solvothermal synthesis	hH-MoS_2	60	14	Assumption of 100% conversion	18
Solvothermal synthesis	MoS_2/RGO	80	1.5	Conversion rate has been given as 90% in the ref	19
Solvothermal synthesis	MoS_2	120	47	Assumption of 100% conversion	20
Solvothermal synthesis	MoS_2/Mo_2C	1000	6.4	Assumption of 100% conversion	21

Supplementary Table 2. A comparison of the HER activity of the HC-Mo_2S/Mo_2C catalyst in this work with previously reported data and our tested data of Pt-based catalysts. N/A stands for Not Available.

Catalysts	Loading mass ($mg\ cm^{-2}$)	Electrolyte	Potential @ 1000 $mA\ cm^{-2}$ (mV), w/o *iR* corr.	η @ 1000 mA cm^{-2} (mV), with *iR* corr.	$\Delta\eta/\Delta\log\|j\|$ @ 1000 $mA\ cm^{-2}$	Ref.
HC-Mo_2S/Mo_2C	10	0.5 M H_2SO_4	455	412	91.95 ± 2.92	This work
HC-Mo_2S/Mo_2C	10	1.0 M KOH	501	441	100.6 ± 9.98	This work
20 wt% Pt/C	2	0.5 M H_2SO_4	500	400	237.70 ±14.24	This work
20 wt% Pt/C	2	1.0 M KOH	555	415	234.81 ± 4.41	This work
20 wt% Pt/C	0.5	0.5 M H_2SO_4	622	511	364.16 ± 25.08	This work
20 wt% Pt/C	0.2	0.5 M H_2SO_4	763	658	657.55 ±140.72	This work
Pt mesh	N/A	0.5 M H_2SO_4	1074	958	1551.72 ±285.48	This work
Pt foil	N/A	0.5 M H_2SO_4	~1220	~1170	1141.95 ± 338.85	This work
40 wt% Pt/C	N/A	0.5 M H_2SO_4	N/A	1107	4365.02 ±505.02	22
20 wt% Pt/C	N/A	0.5 M H_2SO_4	980	400	528.22 ± 78.14	23
20 wt% Pt/C/Nafion	0.2	0.5 M H_2SO_4	N/A	670	N/A	21
Pt foil	N/A	0.5 M H_2SO_4	N/A	660	2035.34 ± 567.94	21
Pt foil	N/A	1.0 M KOH	N/A	822	702.80 ± 28.55	21
Pt wire	N/A	1.0 M KOH	N/A	388	676.81 ± 31.98	24

Supplementary Table 3. A comparison of the high-current-density HER performance of the catalysts in this work with previously reported data of non-noble metal catalysts. N/A stands for Not Available.

Catalysts	Loading mass (mg cm^{-2})	Electrolyte	Potential @ 1000 mA cm^{-2} (mV), w/o *iR* corr.	η @ 1000 mA cm^{-2} (mV), with *iR* corr.	Ref.
HC-Mo_2S/Mo_2C	10	0.5 M H_2SO_4	455	412	This work
HC-Mo_2S/Mo_2C	10	1.0 M KOH	501	441	This work
α-MoB_2	N/A	0.5 M H_2SO_4	790	334	23
LiCoBPO/NF	1	1.0 M KOH	N/A	400	25
NaCoBPO/NF	1	1.0 M KOH	N/A	520	25
Ni_2P	7.4	1.0 M KOH	N/A	620	24
$Ni_{2(1-x)}Mo_{2x}P$	7.4	1.0 M KOH	N/A	300	24
MoS_2/Mo_2C	0.3	0.5 M H_2SO_4	600	250	21
MoS_2/Mo_2C	0.3	1.0 M KOH	420	220	21
Co-Co_2P@NPC/rGO	N/A	0.5 M H_2SO_4	900	N/A	26

Supplementary Table 4. Calculations of H_2 production rate in Fig. 4 in the main text, taken 1 Amper (1A) as an example.

	Parameters	Operating	Answer
1	Current value (Amper, A)	1	1
2	Quantity of electric charge (C s^{-1})	×1	1
3	Quantity of electrons (s^{-1})	× (6.25×10^{18})	6.25×10^{18}
4	Quantity of as-prepared H_2 (s^{-1})	÷2	3.13×10^{18}
5	H_2 mol amount (mol s^{-1})	÷ (6.02×10^{23})	5.2×10^{-6}
6	H_2 mass amount (g s^{-1})	×2	1×10^{-5}
7	H_2 mass amount (mg h^{-1})	× (3600 ×1000)	37.4

Supplementary Table 5. A comparison of the prices of catalysts in this work compared to commercial electrocatalysts for HER in acidic media.

Catalysts	Materials	Suppliers	Price (US$)	Price per 1 m^2 catalyst (US$)
HC-Mo_2S/Mo_2C (This work)	Molybdenite concentrates	China Molybdenum Co., Ltd.	0.26 kg^{-1}	10 (10 mg cm^{-2}, w/o support) in total
HC-Mo_2S/Mo_2C (This work)	Mo_2C additive	Qinghuangdao ENO High-Tech Material Development Co. Ltd.	40 kg^{-1}	
Supports (This work)	Ni foam	Linyi Gelon LIB Co., Ltd.	30 m^{-2}	30
Supports (This work)	Carbon cloth	Jiangsu Sutong Carbon Fiber Co., Ltd	250 m^{-2}	250
Supports (This work)	Cu foam	Kunshan Guangjiayuan Electronic Tech Co. Ltd.	280 m^{-2}	280
Pt/C	10 wt.% Pt/C powder	Alfa Aesar	36 g^{-1}	360 (1 mg cm^{-2}, w/o support)
Pt/C	20 wt.% Pt/C powder	Alfa Aesar	206 g^{-1}	4120 (2 mg cm^{-2}, w/o support)
Pt black on carbon paper	Pt black 2 mg cm^{-2}	Fuel Cell Store	1.35 cm^{-2}	13,500
Pt black on carbon cloth	Pt black 2 mg cm^{-2}	Fuel Cell store	0.91 cm^{-2}	9,100
Pt/C on carbon cloth	60 wt.% Pt/C 0.5 mg cm^{-2}	Fuel Cell store	0.45 cm^{-2}	4,500
Pt commodity	Pt	Shanghai Gold Exchange	27 g^{-1}	N/A
Raney Ni	>90% Ni <10% Al	Jiangsu Leini Metal Tech. Co., Ltd	50 kg^{-1}	30 (60 mg cm^{-2}, w/o support)
MoS_2 solid lubricant on Mo foil[27]	MoS_2 (30% solid content)	OKS 111, Germany	470 kg^{-1}	47 (10 mg cm^{-2}, w/o Mo support)
MoS_2 solid lubricant on Mo foil[27]	Mo foil support	Luoyang Tuojing Refractory Metal Co., Ltd.	613 m^{-2}	613

Supplementary references

1. National minerals information center. Available from: https://www.usgs.gov/centers/nmic/commodity-statistics-and-information.
2. Liu, Z., et al., Unique domain structure of two-dimensional α-Mo_2C superconducting crystals. *Nano Lett.* **16**, 4243-4250 (2016).
3. Chen, J., et al., Ag@MoS_2 core-shell heterostructure as SERS platform to reveal the hydrogen evolution active sites of single-layer MoS_2. *J. Am. Chem. Soc.* **142**, 7161-7167 (2020).
4. Young, A. P., et al., A semi-empirical two step carbon corrosion reaction model in PEM fuel cells. *J. Electrochem. Soc.* **160**, 381-388 (2013).
5. Gopalakrishnan, D., Damien, D. & Shaijumon, M. M. MoS_2 quantum dot-interspersed exfoliated MoS_2 nanosheets. *ACS nano* **8**, 5297-5303 (2014).
6. Voiry, D., et al., Enhanced catalytic activity in strained chemically exfoliated WS_2 nanosheets for hydrogen evolution. *Nat. Mater.* **12**, 850-855 (2013).
7. Voiry, D., et al., Conducting MoS_2 nanosheets as catalysts for hydrogen evolution reaction. *Nano Lett.* **13**, 6222-6227 (2013).
8. Lukowski, M. A., et al., Enhanced hydrogen evolution catalysis from chemically exfoliated metallic MoS_2 nanosheets. *J. Am. Chem. Soc.* **135**, 10274-10277 (2013).
9. Kong, D., et al., Synthesis of MoS_2 and $MoSe_2$ films with vertically aligned layers. *Nano Lett.* **13**, 1341-1347 (2013).
10. Wang, H., et al., Electrochemical tuning of MoS_2 nanoparticles on three-dimensional substrate for efficient hydrogen evolution. *ACS nano* **8**, 4940-4947 (2014).
11. Yang, J., et al., Ultrahigh-current-density niobium disulfide catalysts for hydrogen evolution. *Nat. Mater.* **18**, 1309-1314 (2019).
12. Wang, D., et al., Hydrothermal synthesis of MoS_2 nanoflowers as highly efficient hydrogen evolution reaction catalysts. *J. Power Sources* **264**, 229-234 (2014).
13. Zhao, Z., et al., Vertically aligned MoS_2/Mo_2C hybrid nanosheets grown on carbon paper for efficient electrocatalytic hydrogen evolution. *ACS Catal.* **7**, 7312-7318 (2017).
14. Zhang, S., et al., Constructing highly oriented configuration by few-layer MoS_2: toward high-performance lithium-ion batteries and hydrogen evolution reactions. *ACS nano* **9**, 12464-12472

(2015).

15. Yu, X. Y., et al., Ultrathin MoS_2 nanosheets supported on n-doped carbon nanoboxes with enhanced lithium storage and electrocatalytic properties. *Angew. Chem. Int. Edit.* **54**, 7395-7398 (2015).

16. Yan, Y., et al., Ultrathin MoS_2 nanoplates with rich active sites as highly efficient catalyst for hydrogen evolution. *ACS Appl. Mater. Inter.* **5**, 12794-12798 (2013).

17. Tang, Y. J., et al., Molybdenum disulfide/nitrogen-doped reduced graphene oxide nanocomposite with enlarged interlayer spacing for electrocatalytic hydrogen evolution. *Adv. Energy Mater.* **6**, 1600116 (2016).

18. Guo, B., et al., Hollow structured micro/nano MoS_2 spheres for high electrocatalytic activity hydrogen evolution reaction. *ACS Appl. Mater. Inter.* **8**, 5517-5525 (2016).

19. Li, Y., et al., MoS_2 nanoparticles grown on graphene: an advanced catalyst for the hydrogen evolution reaction. *J. Am. Chem. Soc.* **133**, 7296-7299 (2011).

20. Xie, J., et al., Controllable disorder engineering in oxygen-incorporated MoS_2 ultrathin nanosheets for efficient hydrogen evolution. *J. Am. Chem. Soc.* **135**, 17881-17888 (2013).

21. Luo, Y., et al., Morphology and surface chemistry engineering toward pH-universal catalysts for hydrogen evolution at high current density. *Nat. Comm.* **10**, 269-277 (2019).

22. Zhu, L., et al., A rhodium/silicon co-electrocatalyst design concept to surpass platinum hydrogen evolution activity at high overpotentials. *Nat. Comm.* 7, 12272-12278 (2016).

23. Chen, Y., et al., Highly active, nonprecious electrocatalyst comprising borophene subunits for the hydrogen evolution reaction. *J. Am. Chem. Soc.* **139**, 12370-12373 (2017).

24. Yu, L., et al., Ternary $Ni_{2(1-x)}Mo_{2x}P$ nanowire arrays toward efficient and stable hydrogen evolution electrocatalysis under large-current-density. *Nano Energy* **53**, 492-500 (2018).

25. Menezes, P. W., et al., Helical cobalt borophosphates to master durable overall water-splitting. *Energ. Environ. Sci.* **12**, 988-999 (2019).

26. Li, G., et al., Cobalt–cobalt phosphide nanoparticles@nitrogen-phosphorus doped carbon/graphene derived from cobalt ions adsorbed saccharomycete yeasts as an efficient, stable, and large-current-density electrode for hydrogen evolution reactions. *Adv. Funct. Mater.* **28**, 1801332 (2018).

27. Kiriya, D., et al., General thermal texturization process of MoS_2 for efficient electrocatalytic hydrogen evolution reaction. *Nano Lett.*, **16**, 4047-4053 (2016).
