# Supplementary material for: High-throughput production of cheap mineral-based two-dimensional electrocatalysts for high-current-density hydrogen evolution
Source: Nat Commun. 2020 Jul 24;11:3724. doi: 10.1038/s41467-020-17121-8 (PMC7381654; doi:10.1038/s41467-020-17121-8)
Supplement: Supplementary file 2 — Peer Review File [file 41467_2020_17121_MOESM2_ESM.docx]

Peer Review Information

**Journal:** Nature Communications

**Manuscript title:** High-Throughput Production of Cheap Mineral-Based Two-Dimensional Electrocatalysts for High-Current-Density Hydrogen Evolution

**Corresponding author name(s):** Prof Bilu Liu

**Editorial notes:**

**none**

**Reviewer comments & decisions:**

**Reviewer comments, first version:**

Reviewer #1 (Remarks to the Author):

The manuscript by Zhang and coworkers describes an exciting development in the production of high-performing inexpensive electrocatalysts for hydrogen evolution. The authors first demonstrated MoS2-based electrocatalysts modified by MoC and deposited in high surface area supports that could reach very high current densities at moderate overpotentials. Then, they expanded their electrocatalysts synthesis methodology to a new set of catalysts obtained from low-purity mineral Molybdenite and demonstrated similar results. These results are certainly important, especially given the practical relevance of implementing simple, inexpensive, and scalable methods for catalyst manufacturing. The materials characterization presented is thorough, and the paper is clear and concise. However, I found the electrochemical characterization to be insufficient and misleading, and in most cases performed under irrelevant conditions as I will explain in my points below. This is unfortunate, given that this exciting development in electrocatalyst manufacturing could find real-world applications if the performance was demonstrated under relevant electrolysis conditions, and the comparison with Pt was performed under equivalent conditions (i.e., best possible performance) rather than choosing a Pt catalysts morphology that is evidently inferior (i.e., low surface area, flat foils) to the one used for MoS2 (i.e., high-surface area, optimized supports). Given this fact, I am unable to recommend this manuscript for publication in its current form, and would encourage the authors to expand their electrochemical characterization as suggested in the points below.

1. The reason for the vast difference in performance between MoS2 and Pt at high current density doesn’t appear to be associated with the material itself, but rather its morphology. In the supporting information, the authors describe that the double-layer capacitance for their electrocatalysts is128 mF cm-2 vs 0.96 mF cm-2 for Pt. This difference of roughly 2-orders of magnitude in surface area would result in approximately 2-orders of magnitude difference in current density even if the materials were exactly the same – and thus the comparison that is used to motivate the advances presented in the paper is misleading. The authors should avoid presenting results in misleading ways, and should clearly explain this discrepancy in the main manuscript (e.g., presenting the current densities against ECSA rather than project area)

2. Cu supports are inappropriate as they may dissolve under sulfuric acid electrolytes and hence render the exciting results presented irrelevant. I would encourage the authors to use an appropriate support for the electrolysis conditions chosen (e.g., C or Ti) even if their current densities/overpotentials suffer. Otherwise, it is impossible to assess the relevance of the results.

3. All the results presented are based on a 3-electrode cell without iR compensation. This makes it very difficult to understand where the source of losses come from, especially when comparing different electrocatalysts. For example, the linear increase in overpotential with current density for the Pt sample would indicate that the performance is ohmically limited by the transport of ions (or electrons). Such linear behavior is not observed in the MoS2 samples, which would suggest that the cell geometry or positioning of the electrodes was sufficiently different between the two runs to cause changes in the ohmic losses. Given that the authors are studying the electrocatalyst behavior (and not the cell design), it would be much more relevant to report the iR compensated potentials for all the samples so that a fair comparison can be made.

4. Figure 3(g): the overpotential for the two current densities presented is missing.

5. Pt-based electrocatalysts are implemented in membrane-electrode-assembly (MEA) morphologies and have been demonstrated to achieve current in excess of several A/cm2, with minimal overpotentials (<200 mV). This is due to the fact that in MEA morphologies, the catalyst exposure and surface area is maximized to provide the best possible performance. Any comparison with Pt-based electrocatalysts should be either made (1) against optimized MEA electrolyzers or (2) same controlled geometries should be used for both Pt and MoS2 to understand fundamentally the difference between the two electrocatalysts. Otherwise, the results would not be comparing the two electrocatalysts, but rather the difference in the electrochemical cell architecture, and may mislead the community specially if published in a high-impact journal like Nature Communications.


Reviewer #2 (Remarks to the Author):

I like the manuscript by Zhang et al. It addresses a bridge between the well studied MoS2 for HER and its implementation at an industrial scale. While the contribution is very important in that sense, I do have issues with the contribution.
1) The article, while important, does not add any new scientific insight into MoS2. The achievement by the authors is more in the area of materials processing.

2) I like that the author's show that the MoS2 mineral can be used as the catalytic phase after mechanical exfoliation. It would be useful to know what the chemical composition of the mineral is (contaminant levels etc..)

3) I am not sure what the use of the molybdenum carbide is. It decorates the edges and is it only useful for conductivity. If so, would making an ink with carbon black work.

4) I am surprised that the HC-MoS2/Mo2C shows better performance than Pt at high current density. Can the authors speculate on this?

5) It might be useful to move the pH14 data from the SI onto the main text.

**Author rebuttal, first version:**

**Response to Reviewer #1**

**Comment.** The manuscript by Zhang and coworkers describes an exciting development in the production of high-performing inexpensive electrocatalysts for hydrogen evolution. The authors first demonstrated MoS2-based electrocatalysts modified by MoC and deposited in high surface area supports that could reach very high current densities at moderate overpotentials. Then, they expanded their electrocatalysts synthesis methodology to a new set of catalysts obtained from low-purity mineral Molybdenite and demonstrated similar results. These results are certainly important, especially given the practical relevance of implementing simple, inexpensive, and scalable methods for catalyst manufacturing. The materials characterization presented is thorough, and the paper is clear and concise. However, I found the electrochemical characterization to be insufficient and misleading, and in most cases performed under irrelevant conditions as I will explain in my points below. This is unfortunate, given that this exciting development in electrocatalyst manufacturing could find real-world applications if the performance was demonstrated under relevant electrolysis conditions, and the comparison with Pt was performed under equivalent conditions (i.e., best possible performance) rather than choosing a Pt catalysts morphology that is evidently inferior (i.e., low surface area, flat foils) to the one used for MoS2 (i.e., high-surface area, optimized supports). Given this fact, I am unable to recommend this manuscript for publication in its current form, and would encourage the authors to expand their electrochemical characterization as suggested in the points below.

**Response.** Thank you very much for the positive recommendations. We appreciate the Reviewer by writing that our results are “exciting” and “important”. Following the Reviewer’s suggestions, we have done more electrochemical characterization. Please see below for details.

**Comment 1.** The reason for the vast difference in performance between MoS2 and Pt at high current density doesn’t appear to be associated with the material itself, but rather its morphology. In the supporting information, the authors describe that the double-layer capacitance for their electrocatalysts is128 mF cm^-2^ vs 0.96 mF cm^-2^ for Pt. This difference of roughly 2-orders of magnitude in surface area would result in approximately 2-orders of magnitude difference in current density even if the materials were exactly the same – and thus the comparison that is used to motivate the advances presented in the paper is misleading. The authors should avoid presenting results in misleading ways, and should clearly explain this discrepancy in the main manuscript (e.g., presenting the current densities against ECSA rather than project area).

**Response 1.** This is a very good point. We apologize for not writing it clearly in the previous manuscript. To avoid misleading, we have taken your suggestions and added two results in the revised version. First, we agree with the Reviewer that the intrinsic activity of Pt is better than HC-MoS2/Mo2C (normalized by ECSA, Figure R1). Second, we carried out morphology engineering of Pt catalysts and used the data from the best Pt catalyst in the revised version for a fair comparison with HC-MoS2/Mo2C. Specifically, we prepared five types of Pt catalysts with different morphology and ECSA, including Pt foil, Pt mesh, and three Pt/C catalysts loaded on high-surface-area Cu foams with loading mass of 0.2, 0.5 and 2 mg cm^-2^ (Figure R2). Among them, the 2 mg cm^-2^ Pt/C on Cu foam with a large ECSA of 1434 cm^2^ was the best and was chosen for comparison. We find that the HC-MoS2/Mo2C is equally good to this best Pt (412 mV@ 1000 mA cm^-2^ for MoS2 and 400 mV@1000 mA cm^-2^ for the best Pt, Figure S15a on Page 14 in SI). We also note that our optimized Pt performance was close to the best performance of Pt catalysts reported in literature so far (388 mV @ 1000 mA cm^-2^, which is the lowest

3

number we found via careful survey of many literature, see Table S2 on Page 28 in SI). We accordingly changed all the data of Pt foil into those of 2 mg cm^-2^ Pt/C in the revised version. Note that the performance of MoS2 is comparable to best Pt at high current density, while the cost of the MoS2 is much lower than Pt.

**Changes to the revised manuscript.** We rewrote three sentences on Pages 8 and 9 per your comment, “The HER performance of catalysts was compared by the overpotential (*η*) as given in the following equation”, “Compared to the best Pt sample with morphology engineering, *i.e.*, Pt/C loaded on high-surface-area Cu foam with a loading mass of 2 mg cm^-2^, the HC-MoS2/Mo2C needed a larger overpotential to obtain an identical current density (*j*) at small current densities, suggesting that it has a lower intrinsic activity than Pt (Figures S15 and S16)”, and “As current density increased to over 400 mA cm^−2^, the HC-Mo2S/Mo2C required comparable overpotentials to obtain the same current density to the Pt-based catalysts (Figure S17 and Table S2). For instance, the HC-MoS2/Mo2C needed 412 mV @ 1000 mA cm*^−^*^2^, while the respective values for 0.5 mg cm^-2^ Pt/C and 2 mg cm^-2^ Pt/C were 511 mV and 400 mV”.

-0.6	-0.4	-0.2	0.0

HC-MoS_2_/Mo_2_C

Pt/C (2 mg cm^-2^)

Current density (mA cm-2ECSA)

0.0

-0.5

-1.0

Potential **(**V *vs* RHE**)**

Figure R1. ECSA normalized activities of HC-MoS2/Mo2C and 2 mg/cm^2^ Pt/C with *iR* correction. This figure was added as Figure S15b in the revised SI.

Current density (mA cm-2)

-1000

-1.2	-0.8	-0.4	0.0

-250

-500

-750

0

Pt foil

HC-MoS_2_/Mo_2_C

Pt/C (0.2 mg cm^-2^) Pt/C (0.5 mg cm^-2^) Pt/C (2 mg cm^-2^) Pt mesh

Potential **(**V *vs* RHE**)**

Figure R2. Polarization curves of HC-MoS2/Mo2C and different Pt catalysts with *iR* correction. This figure was added as Figure S17b in the revised SI.

**Comment 2.** Cu supports are inappropriate as they may dissolve under sulfuric acid electrolytes and hence render the exciting results presented irrelevant. I would encourage the authors to use an appropriate support for the electrolysis conditions chosen (e.g., C or Ti) even if their current densities/overpotentials suffer. Otherwise, it is impossible to assess the relevance of the results.

4

**Response 2.** Thank you for this comment. You are right that Cu may dissolve in sulfuric acid when Cu is oxidized, but the dissolution is slow. In our experimental conditions, we found that the dissolution of Cu was negligible and did not contribute to the catalytic performance. First, the polarization curves of the HC-MoS2/Mo2C before and after subtracting Cu background signal nearly overlap each other (Figure R3). Second, we measured the Faradaic efficiency of HC-MoS2/Mo2C on Cu to be nearly 100%, further suggesting the negligible dissolution of Cu in our experiment conditions (Figure R4). These results showed that the measured performance was reliable.

Following your suggestion, we also shown the performance of HC-MoS2/Mo2C on other supports like Ti and carbon cloth (Figure S3, Page 4 in SI).

**Changes to the revised manuscript.** We added two sentences on Page 9, “The dissolution of Cu in sulfuric acid and the effect of Cu support on current densities in HER is negligible in our experiments (Figure S14)” and “Moreover, the HC-MoS2/Mo2C gives a nearly 100% Faradaic efficiency during HER (Figure S18)”.

Potential (V *vs* RHE)

-0.4	-0.3	-0.2	-0.1	0.0

Current density (mA cm^-2^)

-1000

-250

-500

-750

0

-1000

-600

-800

-0.41	-0.40	-0.39

Enlarged view

With Cu correction W/o Cu correction

Figure R3. Polarization curves of the HC-MoS2/Mo2C before and after correcting Cu background signal. This figure was added as Figures S14b in the revised SI.

Time (min)

0 5 10 15 20 25 30

H_2_ produced (mL)

40

50

30

20

10

0

Calculated

Measured

40

80

60

20

0

100

Faradaic efficiency (%)

Figure R4. Faradaic efficiency test by measuring H2 volume using water drainage method. This figure was added as Figures S18b in the revised SI.

**Comment 3.** All the results presented are based on a 3-electrode cell without iR compensation. This makes it very difficult to understand where the source of losses come from, especially when comparing different electrocatalysts. For example, the linear increase in overpotential with current density for the Pt sample would indicate that the performance is ohmically limited by the transport of ions (or electrons). Such linear behavior is not observed in the MoS2 samples, which would suggest that the cell geometry or positioning of the electrodes was sufficiently different between the two runs to cause

5

changes in the ohmic losses. Given that the authors are studying the electrocatalyst behavior (and not the cell design), it would be much more relevant to report the iR compensated potentials for all the samples so that a fair comparison can be made.

**Response 3.** Thanks for pointing out this issue, which we didn’t write clearly in our previous manuscript. First, all the electrochemical characterization was conducted by using the same cell and same test parameters (Figure R5a and R5b). The height and diameter of the cylindrical cell are 9 cm and 5 cm, respectively. During all the tests, a working electrode (WE, no matter HC-MoS2/Mo2C or Pt) is in the middle of the cell and a reference electrode (RE) and a counter electrode (CE) are on both sides. The RE is connected with a Luggin capillary towards the WE. The distance between the WE and the RE is fixed to keep the solution resistances same in different runs, verified by the EIS results as shown in Figure R5c. Second, according to your suggestions, we have put the *iR* corrected data in Figures 3a, 3c, and 4c in the revised manuscript. We also put all raw data without *iR* corrections in the revised SI to give comprehensive information. Third, the different behaviors between HC-MoS2/Mo2C and Pt foil stem from their different chemical compositions and morphologies, rather than the structure of the electrolytic cell. The poor mass transfer ability and the small number of active sites on Pt foil may contribute to its linear increase in overpotential with current density. The data shown in Figure R2 also support this point.

**Changes to the revised manuscript.** To show the identical test conditions, we added a sentence on Page 8, “All the electrochemical characterization was conducted using the same cell and the same test parameters (Figure S9)”.

RE	WE CE

Luggin Capillary

CE WE

RE

1 cm

1 cm

**a	b**

150

**c**

100

2

Enlarged view

50

1

0

1

0

-Z" (Ω)

HC-MoS_2_/Mo_2_C 2D MoS_2_

Pt/C (2 mg cm^-2^) Cu foam

0	50	100	150	200

**Z' (Ω)**

Figure R5. (a,b) Top-view and side-view photos of the cell for performing electrochemical tests. (c) EIS curves of different catalysts in a 0.5 M H2SO4 solution. These figures were added as Figures S9 and S22 in the revised SI.

**Comment 4.** Figure 3(g): the overpotential for the two current densities presented is missing. **Response 4.** We have added the potential values in Figure 3g in the revised manuscript.

**Comment 5.** Pt-based electrocatalysts are implemented in membrane-electrode-assembly (MEA) morphologies and have been demonstrated to achieve current in excess of several A/cm^2^, with minimal overpotentials (<200 mV). This is due to the fact that in MEA morphologies, the catalyst exposure and surface area is maximized to provide the best possible performance. Any comparison with Pt-based electrocatalysts should be either made (1) against optimized MEA electrolyzers or (2) same controlled geometries should be used for both Pt and MoS2 to understand fundamentally the difference between

6

the two electrocatalysts. Otherwise, the results would not be comparing the two electrocatalysts, but rather the difference in the electrochemical cell architecture, and may mislead the community specially if published in a high-impact journal like Nature Communications.

**Response 5.** We totally agree with this nice comment. The catalytic performance mentioned by the Reviewer “...*current in excess of several A/cm^2^, with minimal overpotentials (<200 mV) ...*” is achieved by MEA in anion exchange membrane or proton exchange membrane water electrolyzers, which have very different structures to the three-electrode electrolytic cell we used here. In addition, the realization of this high performance depends not only on the optimization of the catalyst, but also on the high operating temperature (>50 °C or even 90 °C), as shown in previous reports (*Nat. Nanotechnol.* 2019, 14, 1071-1074; *J. Power. Sources.* 2008, 177, 281-285). Because we didn’t use MEA electrolyzers in this study, the option (2) that you mentioned is more suitable. As we wrote in detail in Response 3, we used the same controlled geometries to study Pt and MoS2 catalysts. For Pt, we tested the commercial Pt mesh, Pt black and Pt/C loaded on Cu foam (with different Pt amount and engineered performance). Overall, the MoS2 catalyst shows comparable high-current-density performance with our best Pt, as well as the best Pt reported in many literatures under the same three-electrode cell structure (Table S2, Page 28 in SI).

**Response to Reviewer #2**

**Comment.** I like the manuscript by Zhang et al. It addresses a bridge between the well studied MoS2 for HER and its implementation at an industrial scale. While the contribution is very important in that sense, I do have issues with the contribution.

**Response.** Thank you very much for your positive recommendations.

**Comment 1.** The article, while important, does not add any new scientific insight into MoS2. The achievement by the authors is more in the area of materials processing.

**Response 1.** Thank you for this comment. Although this work is practical application oriented and aims at high-throughput production of cheap electrocatalysts, we believe it does offer scientific insights and contains novel elements in terms following points.

(1) **A novel heterostructure made of MoS2/*α*-Mo2C has been constructed.** After carbonization, we found that many metallic *α*-Mo2C nanocrystals were converted from semiconducting MoS2 and constructed the metallic/semiconducting lateral heterostructures (Figures 2e and S7). We also found that Mo2C components in the HC-MoS2/Mo2C were critical to the pH-universal HER performance and could promote water dissociation dynamics (Figures S20 and S21, Pages 19-20 in SI).
(2) **A soldering phenomenon between MoS2 and support is proposed.** We discovered a new phenomenon between MoS2 and supports called soldering effect, which renders strong interactions between the catalyst and Cu substrate due to interfacial interaction. This soldering effect can improve the robustness of the catalysts during reactions (Figures 3g and S19 on Page 18 in SI), and in principle, should be a general strategy to guide the design of catalysts for other reactions.
(3) **A generalized slope defined as Δ*η*/Δlog|*j*| for evaluating performance of catalyst at broad potential windows.** This slope circumambulates the limitations of Tafel slope which mainly works for small-current reactions. This generalized slope provides new scientific insight in evaluating the overall properties of catalyst at broad potential windows.

7

**Comment 2.** I like that the author's show that the MoS2 mineral can be used as the catalytic phase after mechanical exfoliation. It would be useful to know what the chemical composition of the mineral is (contaminant levels etc.)

**Response 2.** We analyzed the chemical composition of the molybdenite mineral carefully and the detailed data have been shown in Figures S23-25. First, XPS and EDS show that molybdenite contains oxygen (39.0 at%), sulfur (24.4 at%), molybdenum (13.7 at%), silicon (10.7 at%), magnesium (4.4 at%), calcium (3.0 at%), aluminum (1.4 at%), iron (1.4 at%), tungsten (1.2 at%), and sodium (0.9 at%). Second, XRD and Raman results show that molybdenite contains crystalline MoS2, molybdenum oxides (MoO2 and MoO3), and silicates.

**Changes to the revised manuscript.** We added a sentence in the revised manuscript on Page 13, “Detailed characterization indicated that the molybdenite concentrate mainly composed of crystalline MoS2, as well as molybdenum oxides (MoO2 and MoO3), silicates and few other compositions (Figures S23-S25)”.

**Comment 3.** I am not sure what the use of the molybdenum carbide is. It decorates the edges and is it only useful for conductivity. If so, would making an ink with carbon black work.

**Response 3.** Molybdenum carbide (Mo2C) is not only useful for electrical conductivity, but also has functions of assisting exfoliation of MoS2 and enhancing catalytic performance. Specifically, there are two types of Mo2C involved. One is Mo2C microparticle (~44 μm) that works as hard intermediary to assist exfoliation of MoS2 bulks because Mo2C has high hardness. Another type is Mo2C nanocrystals (~2.8 nm) which were generated during thermal treatments of MoS2. It improves the electrical conductivity of catalysts as mentioned by the Reviewer and improves the HER performance. Together, Mo2C has functions of assisting exfoliation of MoS2, increasing electrical conductivity, and enhancing catalytic performance. Carbon black cannot replace Mo2C because it is soft and cannot help exfoliating MoS2 in the first step.

**Comment 4.** I am surprised that the HC-MoS2/Mo2C shows better performance than Pt at high current density. Can the authors speculate on this?

**Response 4.** This is an interesting point. The intrinsic activity of Pt is better than HC-MoS2/Mo2C. At high current density, HC-MoS2/Mo2C is comparable to Pt (Figures 3f, S17 and Table S2). We think this good performance of the HC-MoS2/Mo2C originates from large numbers of active sites, good mass transfer ability and proper microscale morphology, which compensate its relatively low intrinsic activity and leads to a decent high-current performance.

**Comment 5.** It might be useful to move the pH14 data from the SI onto the main text.

**Response 5.** We have taken this suggestion and moved the pH 14 data onto the main text as shown in

Figure 3a.

**Reviewer comments, second version:**

Reviewer #1 (Remarks to the Author):

The authors have addressed all my comments and I recommend the article publication in its current form


Reviewer #2 (Remarks to the Author):

The authors have done a good job in addressing my comments in my initial review. My major issue from before was the level of insight into the working of the catalyst and the authors have addressed my concerns in their responses and in the modified manuscript. I have a bit of an issue with the so-called solder effect. I do not see this as being any different than standard annealing/heat treatment processes to produce better interaction between catalyst and support. The authors should expand on this point so the reader knows why this is novel.

**Author rebuttal, second version:**

**Response to Reviewer #1**

**Comment.** The authors have addressed all my comments and I recommend the article publication in its current form.

**Response.** Thank you very much for the positive recommendations.

**Response to Reviewer #2**

**Comment.** The authors have done a good job in addressing my comments in my initial review. My major issue from before was the level of insight into the working of the catalyst and the authors have addressed my concerns in their responses and in the modified manuscript.

**Response.** Thank you very much for the positive recommendations.

**Comment 1.** I have a bit of an issue with the so-called solder effect. I do not see this as being any different than standard annealing/heat treatment processes to produce better interaction between catalyst and support. The authors should expand on this point so the reader knows why this is novel.

**Response 1.** The soldering effect is realized by the annealing/heat treatment. Soldering effect means the formation of strong interaction at the interface between support and catalysts, while usual annealing/heat treatment does not necessarily lead to this. The soldering effect could not only provide good electrical contact to improve catalytic performance, but also enhance the robustness of the electrode. We explain on this point in the revised manuscript accordingly.

**Changes to the revised manuscript.** We added one sentence on Page 10, “The soldering effect could not only provide good electrical contact to improve catalytic performance but also enhance the robustness of the electrode, which is not a necessary result achieved during usual annealing or heat treatment.”

**Reviewer comments, third version:**

**Author rebuttal, third version:**
